# Supplementary material for: Abietane Diterpenoids from the Bark of Cryptomeria japonica and Their Antifungal Activities against Wood Decay Fungi
Source: Plants (Basel). 2024 Apr 25;13(9):1197. doi: 10.3390/plants13091197 (PMC11085377; doi:10.3390/plants13091197)
Supplement: Supplementary file 1 [file plants-13-01197-s001.zip › plants-2962110-supplementary.pdf]

Supporting Information

Abietane diterpenoids from the bark of *Cryptomeria japonica* and their antifungal activities against wood decay fungi

Chi-I Chang [1,2], Cheng-Chi Chen [3], Sheng-Yang Wang [4,5,†] and Yueh-Hsiung Kuo [6,7,8,*]

[1] Department of Biological Science and Technology, National Pingtung University of Science and Technology, Pingtung 912, Taiwan; changchii@mail.npust.edu.tw
[2] Traditional Herbal Medicine Research Center, Taipei Medical University Hospital, Taipei 110, Taiwan
[3] Department of Chemistry, National Taiwan University, Taipei 106, Taiwan; r93223083@ntu.edu.tw
[4] Department of Forestry, National Chung-Hsing University, Taichung 402, Taiwan
[5] Agricultural Biotechnology Research Center, Academia Sinica, Taipei 115, Taiwan
[6] Department of Chinese Pharmaceutical Sciences and Chinese Medicine Resources, College of Pharmacy, China Medical University, Taichung 404, Taiwan
[7] Department of Biotechnology, Asia University, Taichung 413, Taiwan
[8] Chinese Medicine Research Center, China Medical University, Taichung 404, Taiwan

* Correspondence: kuoyh@mail.cmu.edu.tw; Tel.: +886-4-2205-3366 (ext. 5701); Fax: +886-4-2207-1693
† The author contributed equally to this paper.

Table of Contents

Figure S1. ^{1}H-NMR spectrum of compound **1** in $CDCl_3$
Figure S2. ^{13}C-NMR spectrum and DEPT of compound **1** in $CDCl_3$
Figure S3. HMQC spectrum of compound **1** in $CDCl_3$
Figure S4. HMBC spectrum of compound **1** in $CDCl_3$
Figure S5. ^{1}H-^{1}H COSY spectrum of compound **1** in $CDCl_3$
Figure S6. NOSEY spectrum of compound **1** in $CDCl_3$
Figure S7. IR spectrum of compound **1**
Figure S8. Mass spectrum of compound **1**
Figure S9. UV-Vis spectrum of compound **1**
Figure S10. ^{1}H-NMR spectrum of compound **2** in $CDCl_3$
Figure S11. ^{13}C-NMR spectrum and DEPT of compound **2** in $CDCl_3$
Figure S12. HMQC spectrum of compound **2** in $CDCl_3$
Figure S13. HMBC spectrum of compound **2** in $CDCl_3$
Figure S14. ^{1}H-^{1}H COSY spectrum of compound **2** in $CDCl_3$
Figure S15. NOSEY spectrum of compound **2** in $CDCl_3$
Figure S16. IR spectrum of compound **2**
Figure S17. Mass spectrum of compound **2**
Figure S18. UV-Vis spectrum of compound **2**
Figure S19. ^{1}H-NMR spectrum of compound **4** in $CDCl_3$
Figure S20. ^{13}C-NMR spectrum and DEPT of compound **4** in $CDCl_3$
Figure S21. HMQC spectrum of compound **4** in $CDCl_3$
Figure S22. HMBC spectrum of compound **4** in $CDCl_3$
Figure S23. ^{1}H-^{1}H COSY spectrum of compound **4** in $CDCl_3$
Figure S24. NOSEY spectrum of compound **4** in $CDCl_3$
Figure S25. IR spectrum of compound **4**
Figure S26. Mass spectrum of compound **4**
Figure S27. UV-Vis spectrum of compound **4**
Figure S28. ^{1}H-NMR spectrum of compound **5** in $CDCl_3$
Figure S29. ^{13}C-NMR spectrum and DEPT of compound **5** in $CDCl_3$
Figure S30. HMQC spectrum of compound **5** in $CDCl_3$
Figure S31. HMBC spectrum of compound **5** in $CDCl_3$
Figure S32. ^{1}H-^{1}H COSY spectrum of compound **5** in $CDCl_3$
Figure S33. NOSEY spectrum of compound **5** in $CDCl_3$
Figure S34. IR spectrum of compound **5**
Figure S35. Mass spectrum of compound **5**
Figure S36. UV-Vis spectrum of compound **5**
Figure S37. ^{1}H-NMR spectrum of compound **6** in $CDCl_3$

Figure S38. ^{13}C-NMR spectrum and DEPT of compound **6** in $CDCl_3$
Figure S39. HMQC spectrum of compound **6** in $CDCl_3$
Figure S40. HMBC spectrum of compound **6** in $CDCl_3$
Figure S41. ^{1}H-^{1}H COSY spectrum of compound **6** in $CDCl_3$
Figure S42. NOSEY spectrum of compound **6** in $CDCl_3$
Figure S43. IR spectrum of compound **6**
Figure S44. Mass spectrum of compound **6**
Figure S45. UV-Vis spectrum of compound **6**

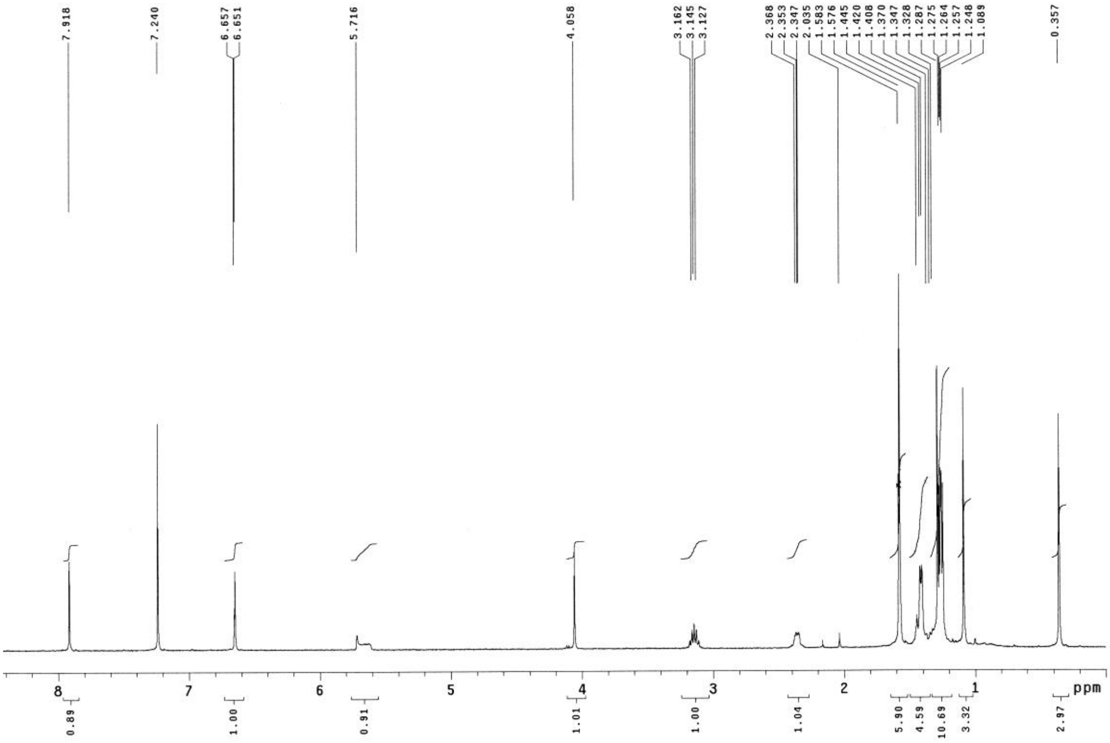

Figure S1. ^{1}H-NMR spectrum of compound **1** in $CDCl_3$

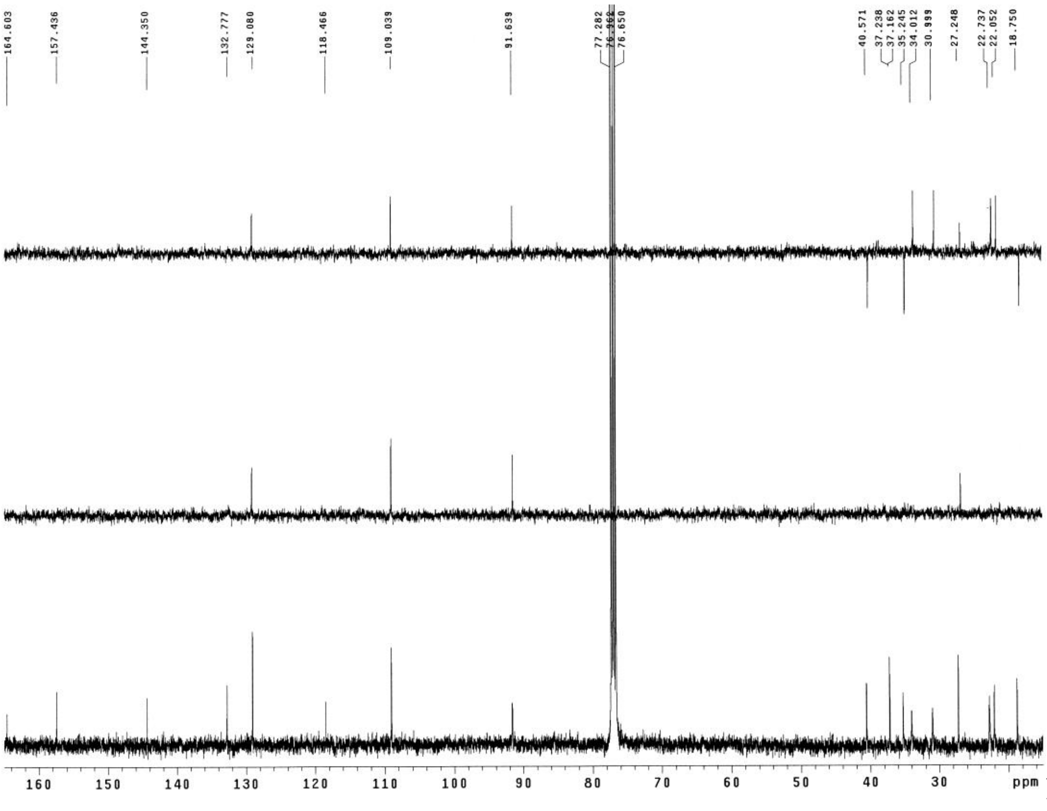

Figure S2. ^{13}C-NMR spectrum and DEPT of compound **1** in $CDCl_3$

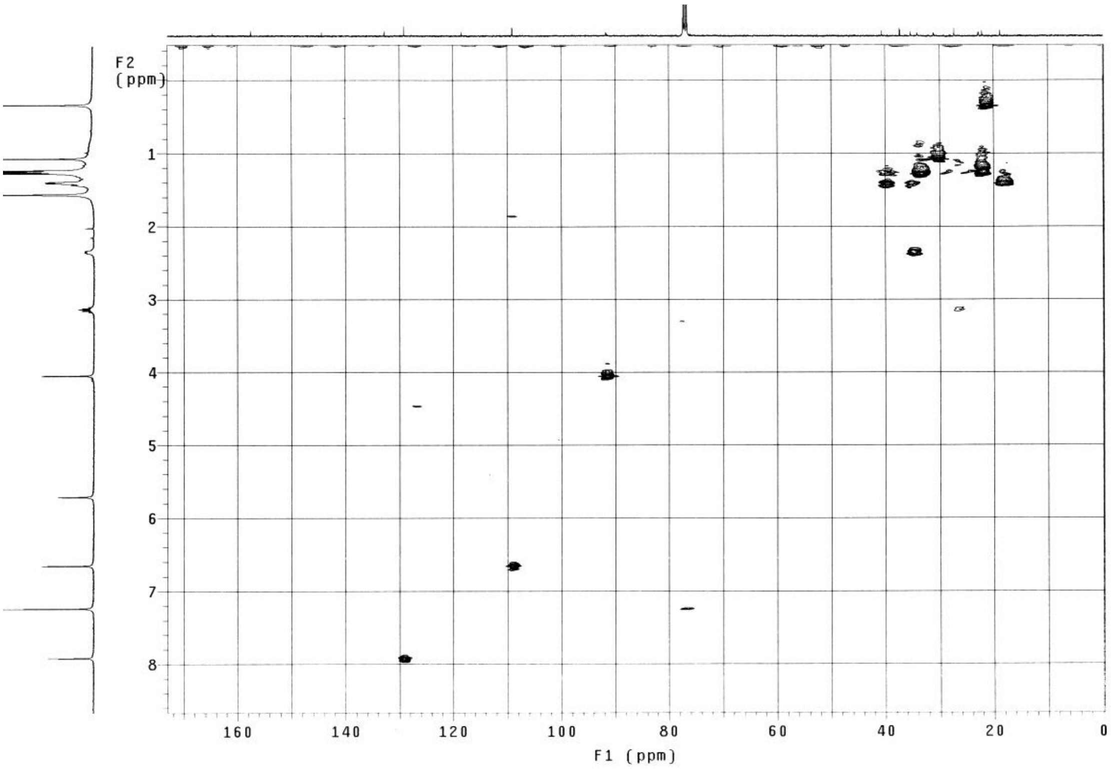

Figure S3. HMQC spectrum of compound **1** in $CDCl_3$

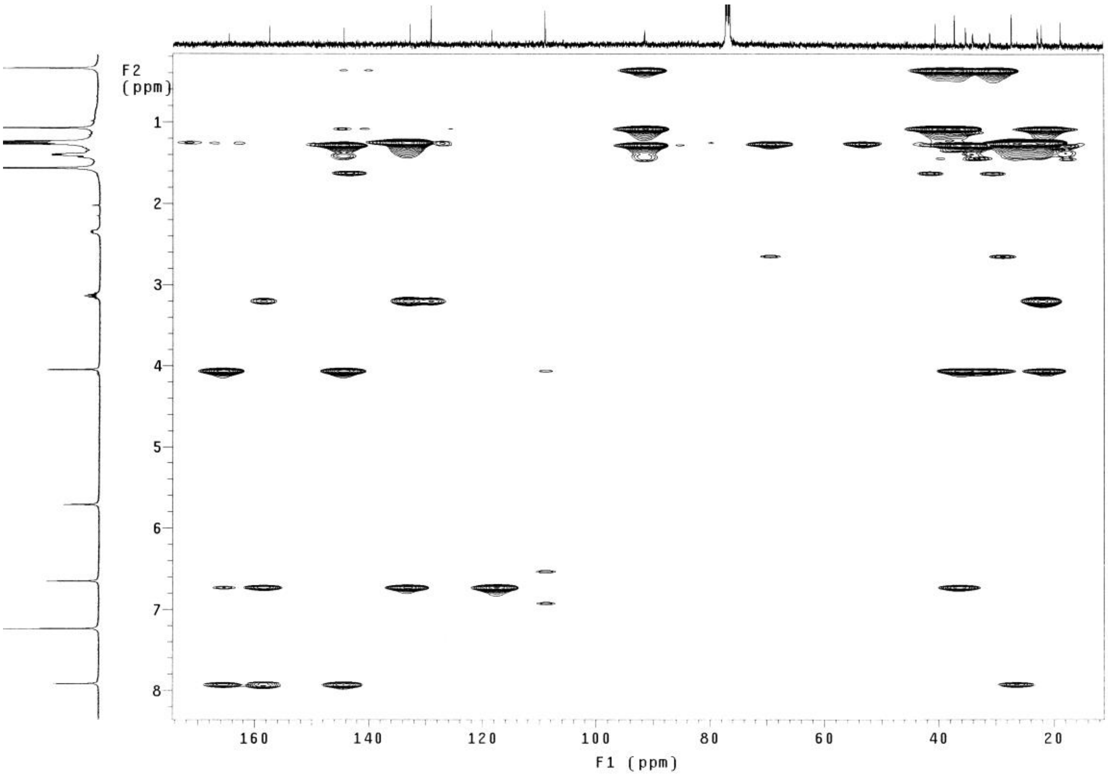

Figure S4. HMBC spectrum of compound **1** in $CDCl_3$

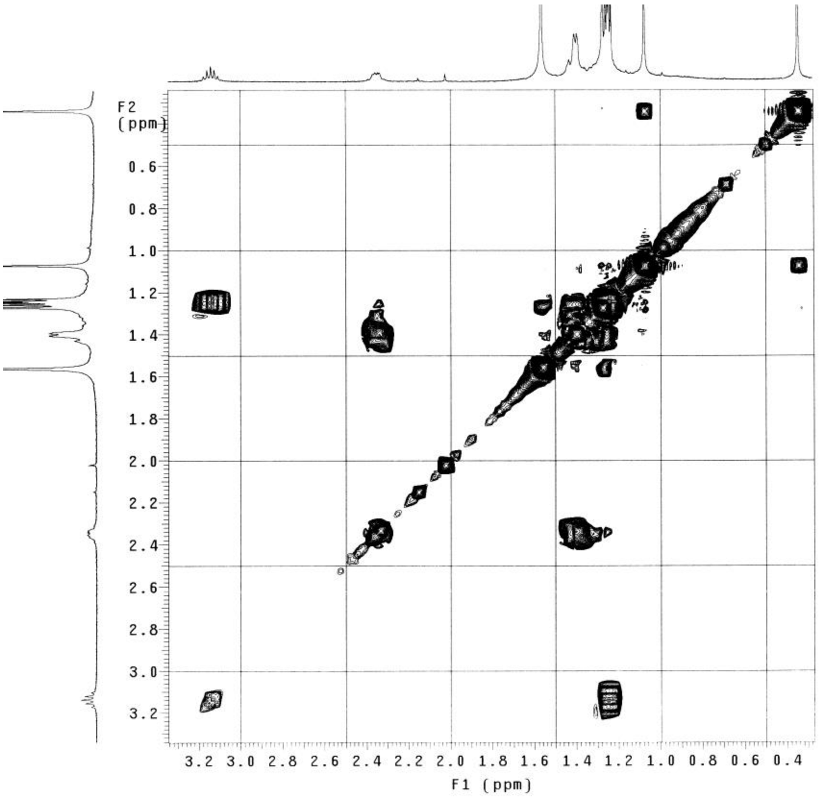

Figure S5. ^{1}H-^{1}H COSY spectrum of compound **1** in $CDCl_3$

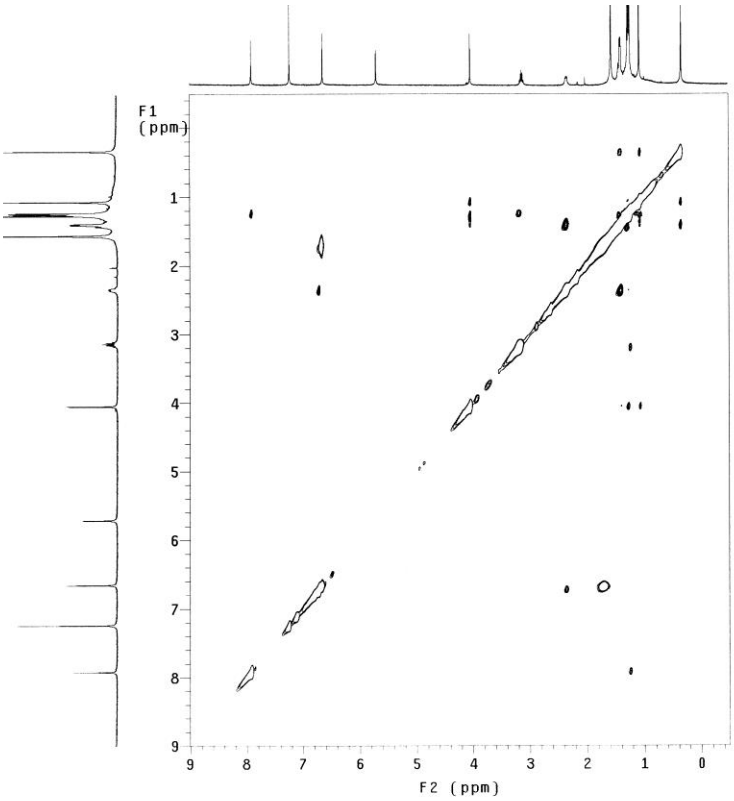

Figure S6. NOSEY spectrum of compound **1** in $CDCl_3$

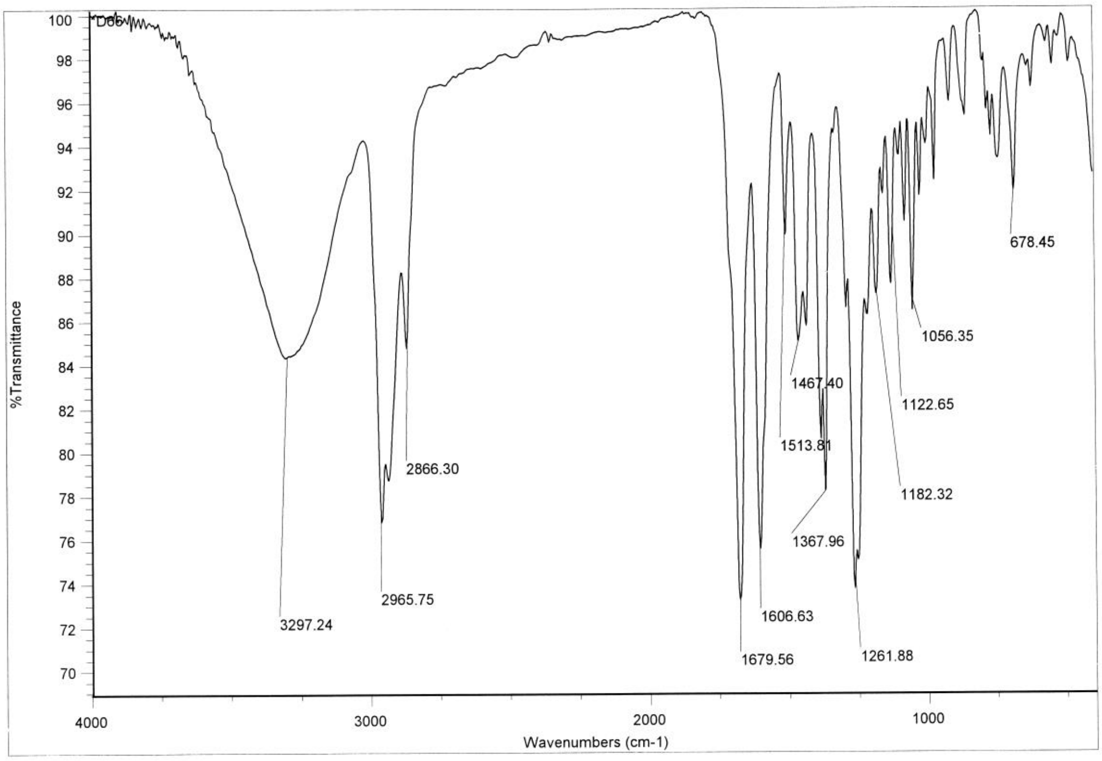

Figure S7. IR spectrum of compound **1**

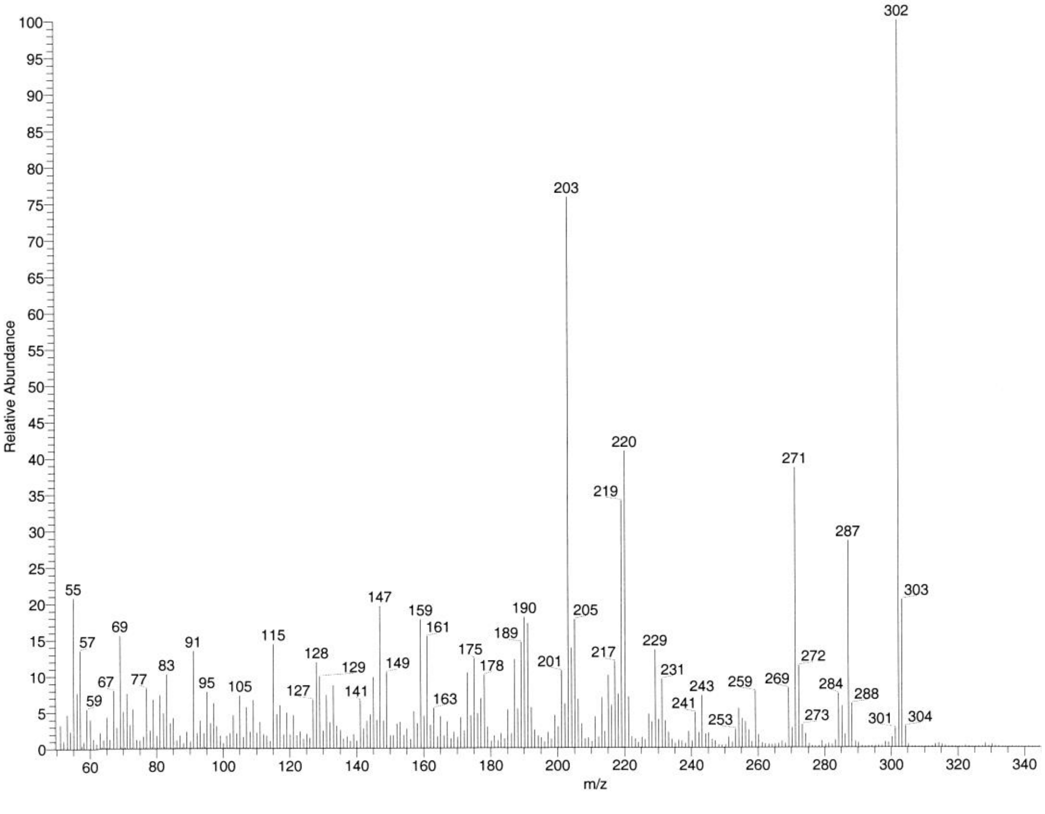

Figure S8. Mass spectrum of compound **1**

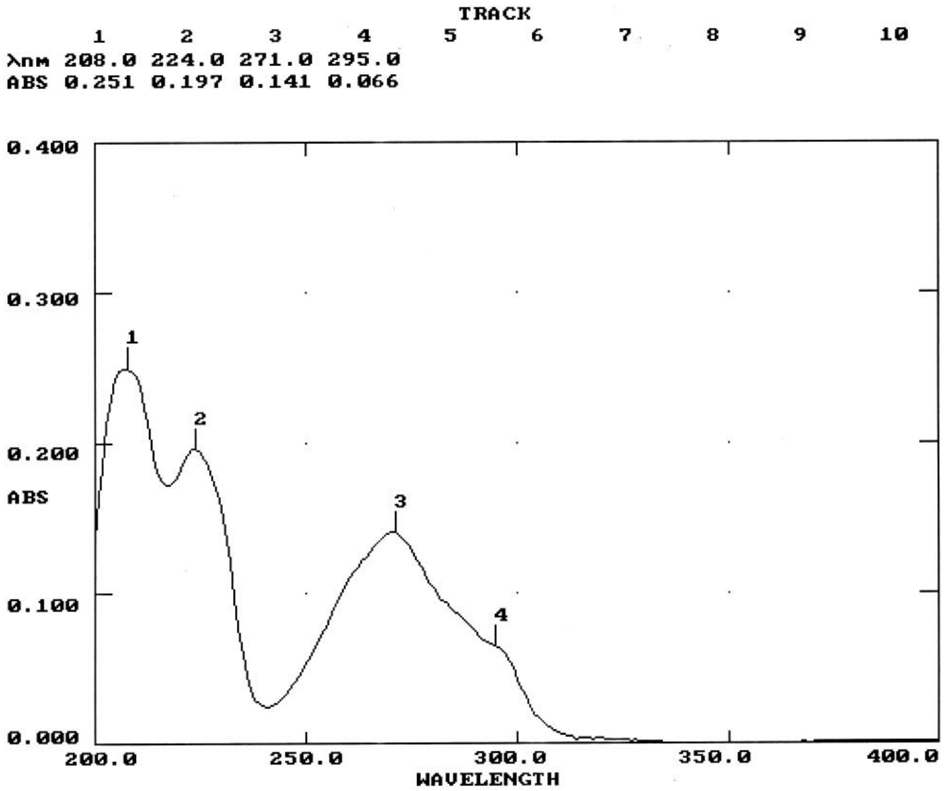

Figure S9. UV-Vis spectrum of compound **1**

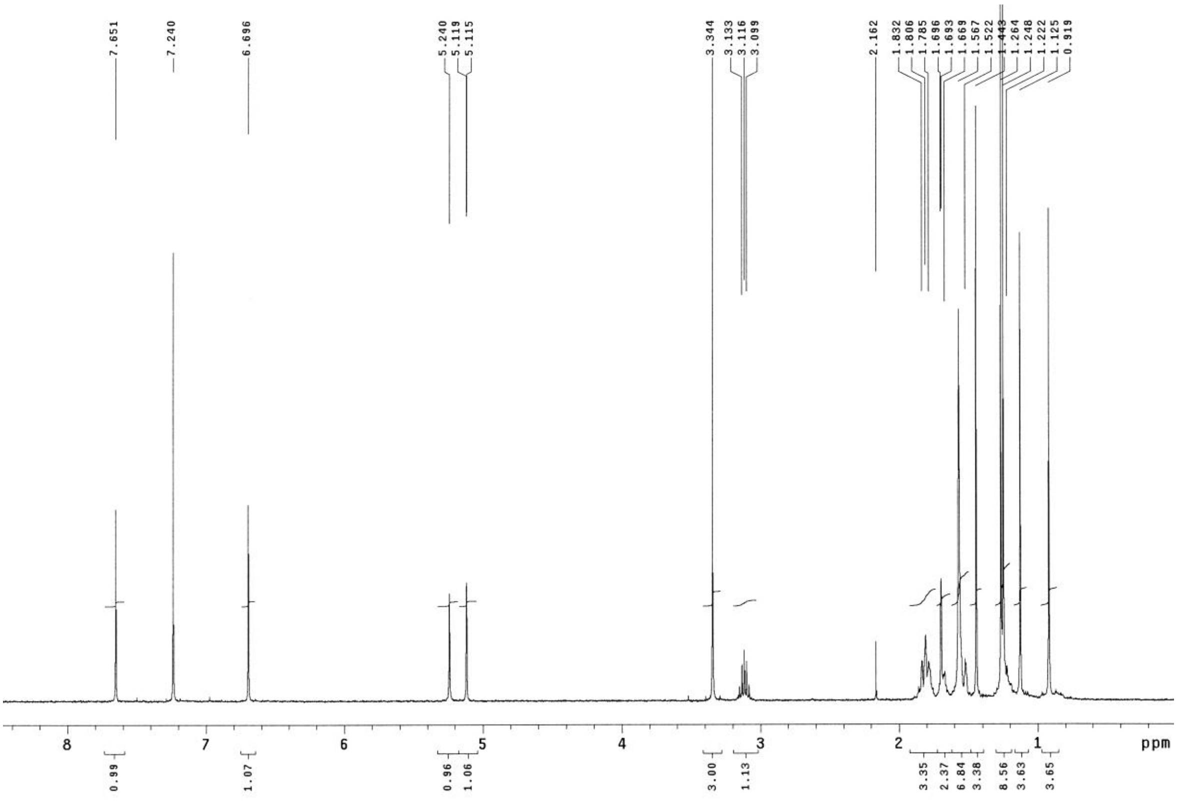

Figure S10. ^{1}H-NMR spectrum of compound **2** in $CDCl_3$

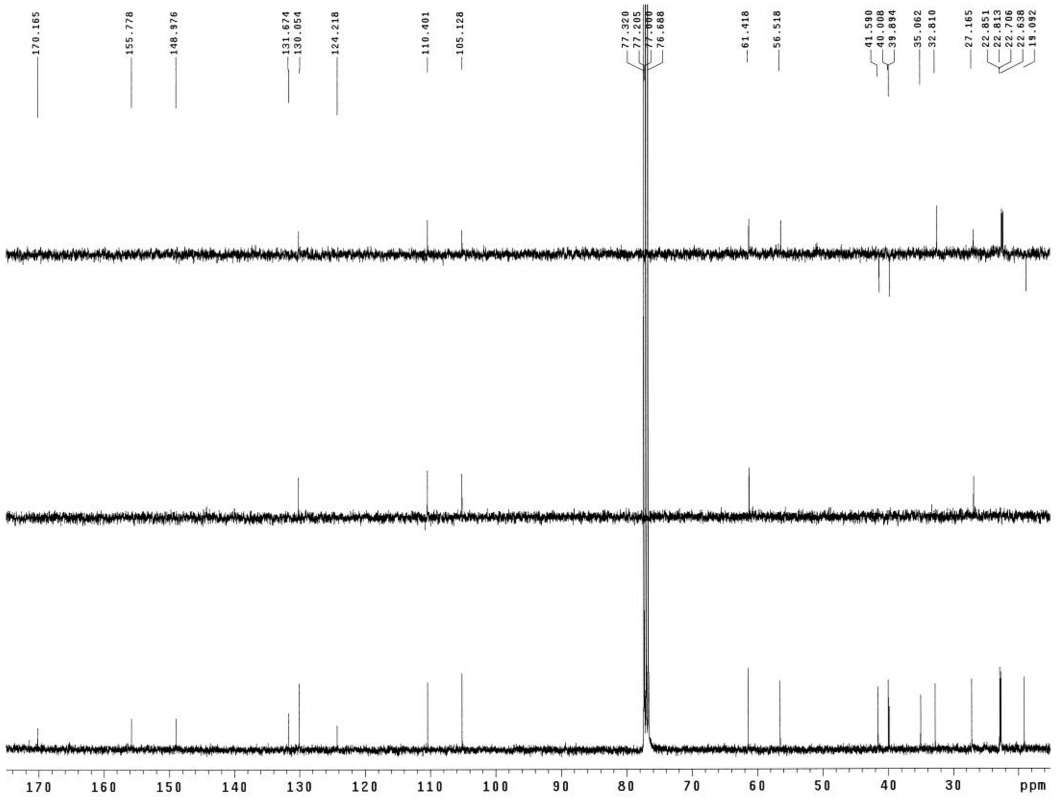

Figure S11. ^{13}C-NMR spectrum and DEPT of compound **2** in $CDCl_3$

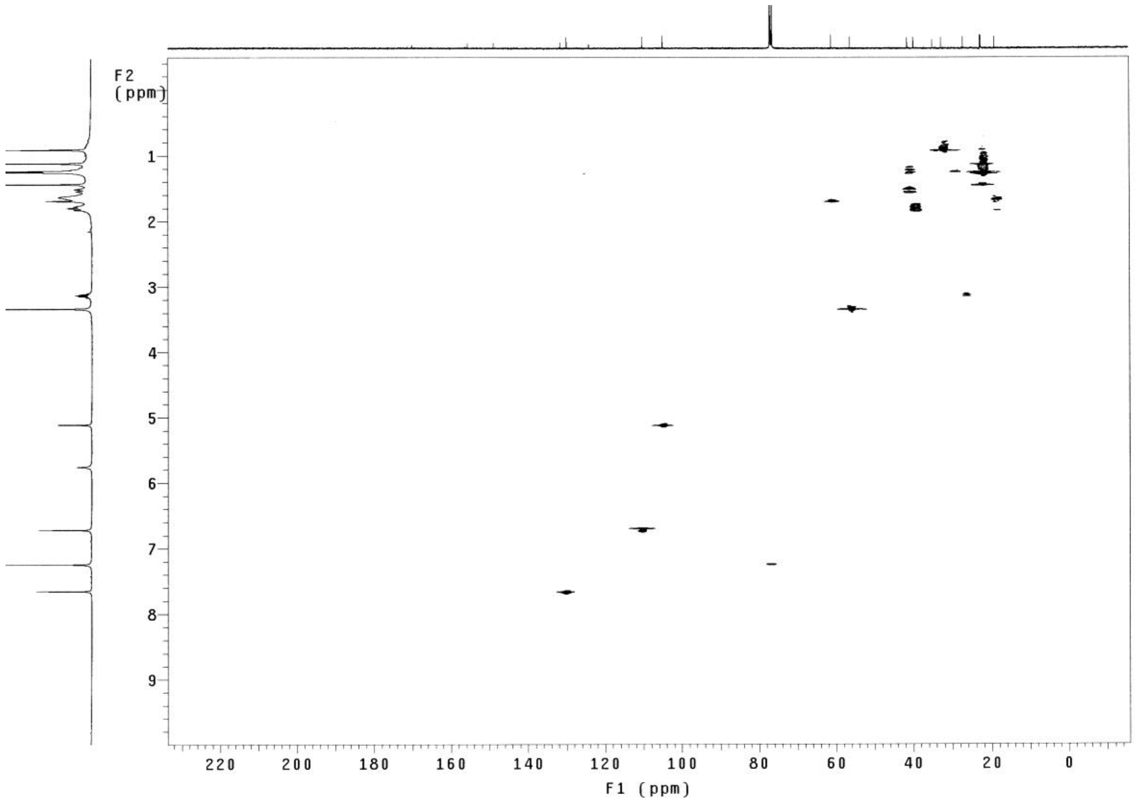

Figure S12. HMQC spectrum of compound **2** in $CDCl_3$

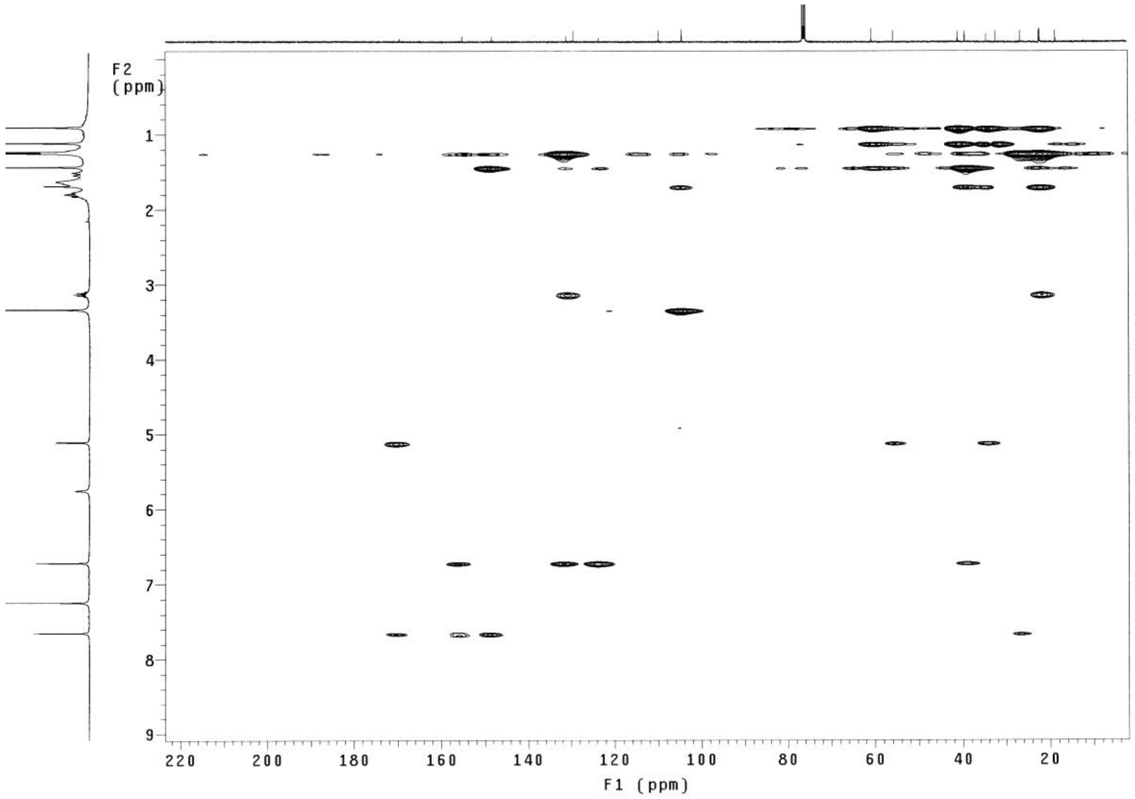

Figure S13. HMBC spectrum of compound **2** in $CDCl_3$

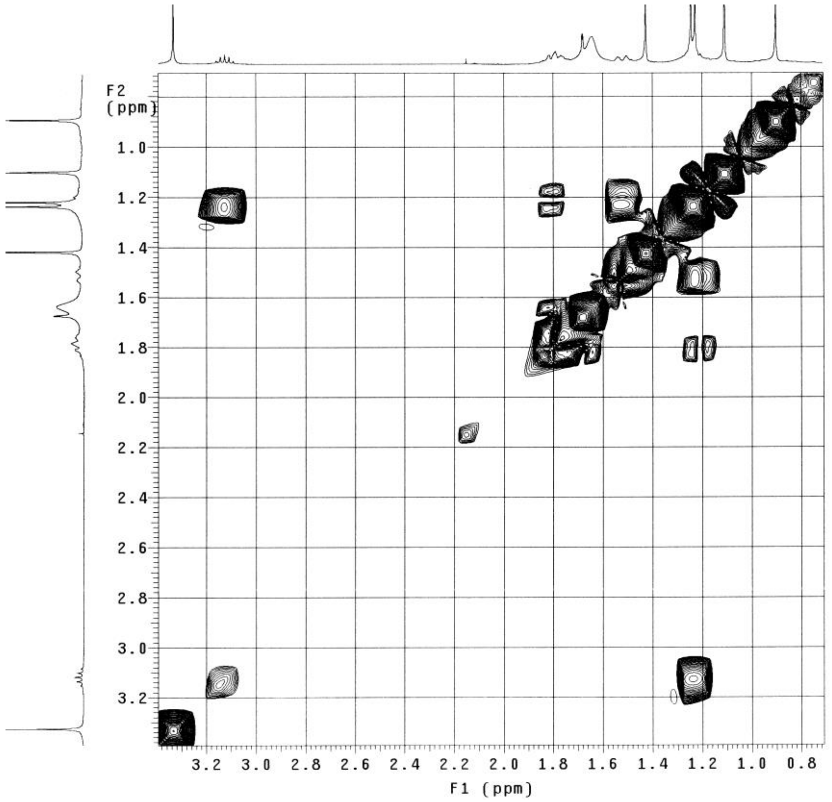

Figure S14. ^{1}H-^{1}H COSY spectrum of compound **2** in $CDCl_3$

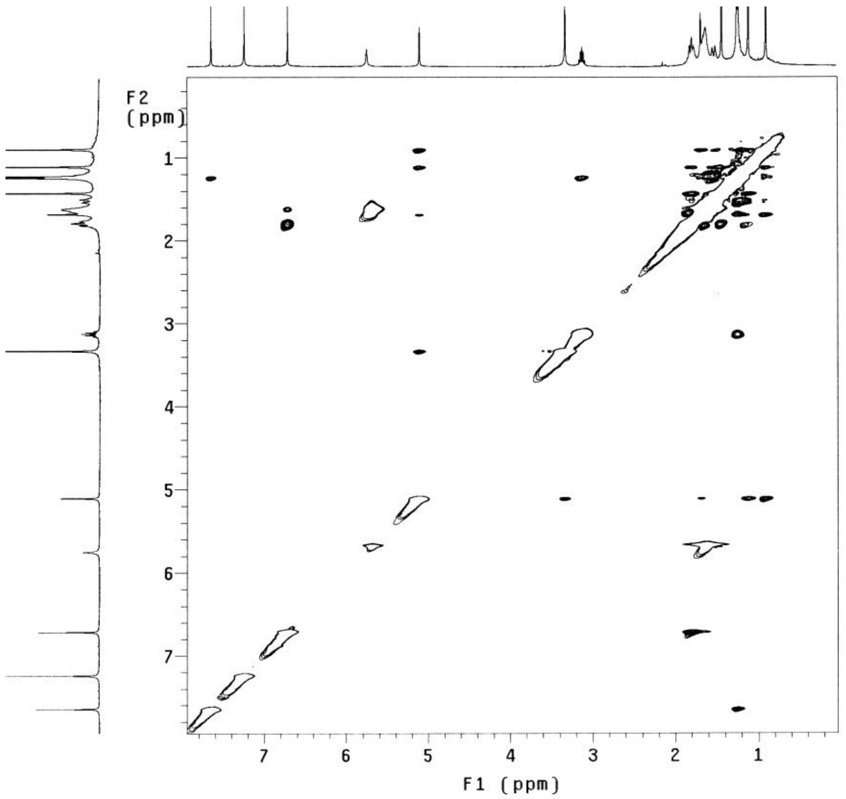

Figure S15. NOSEY spectrum of compound **2** in $CDCl_3$

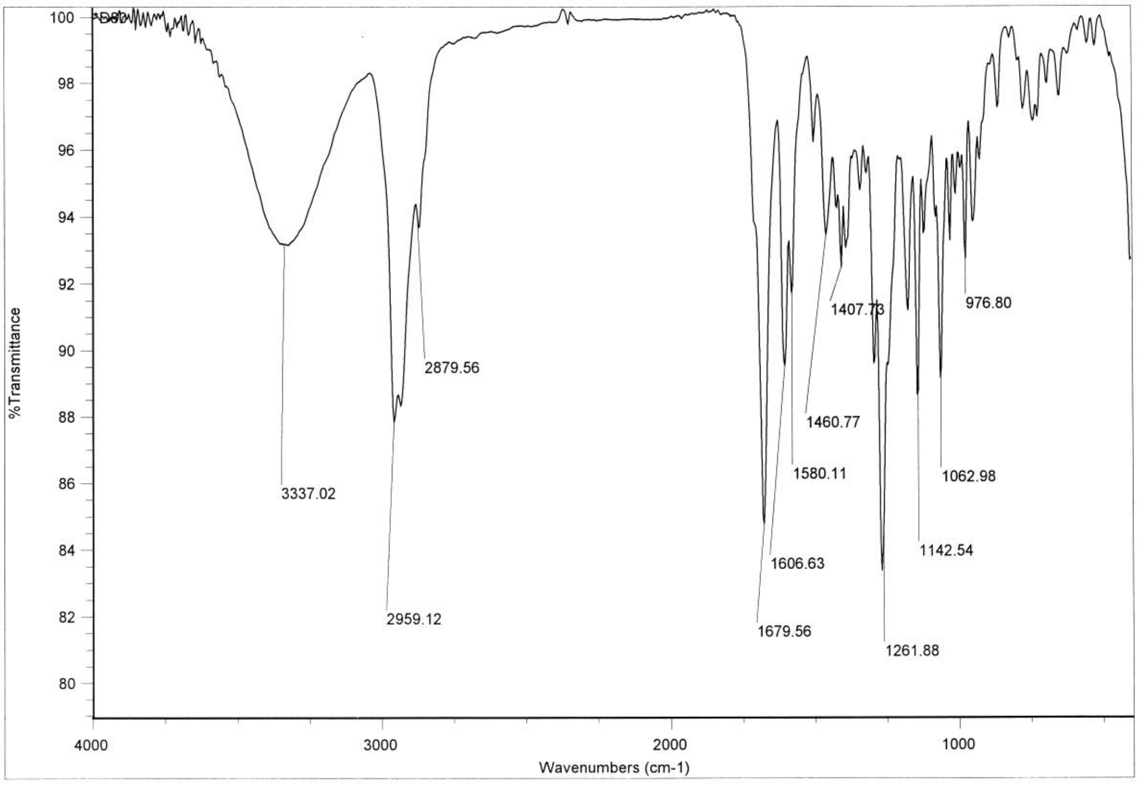

Figure S16. IR spectrum of compound **2**

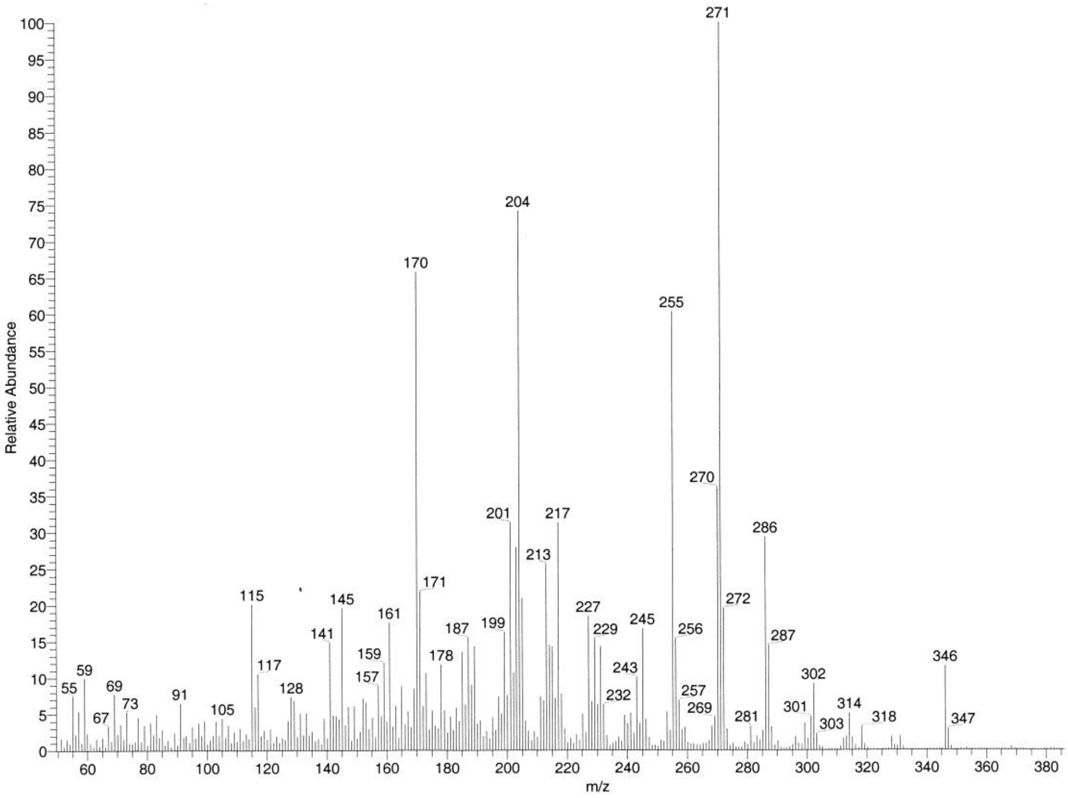

Figure S17. Mass spectrum of compound **2**

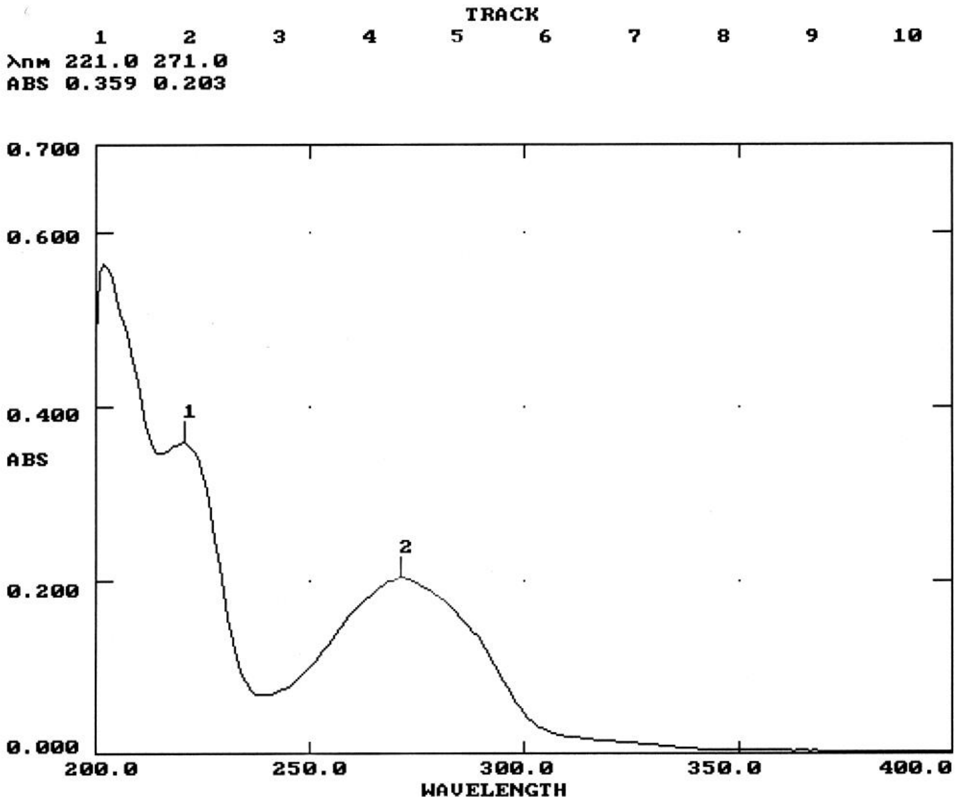

Figure S18. UV-Vis spectrum of compound **2**

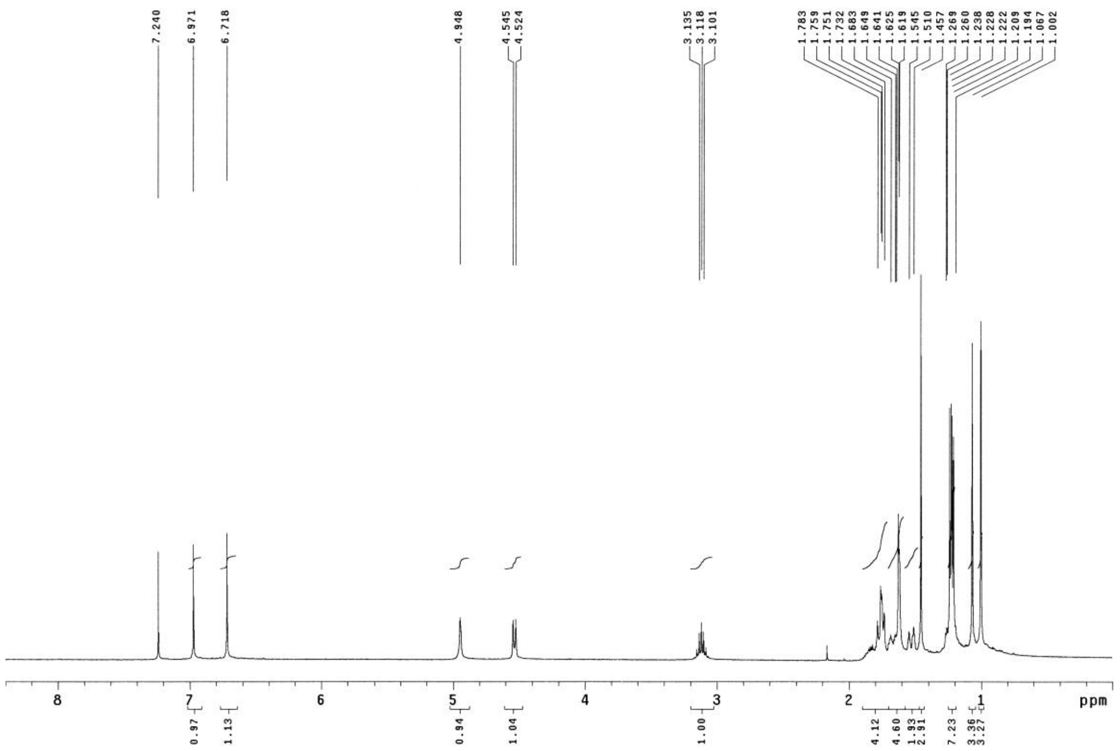

Figure S19. ^{1}H-NMR spectrum of compound **4** in $CDCl_3$

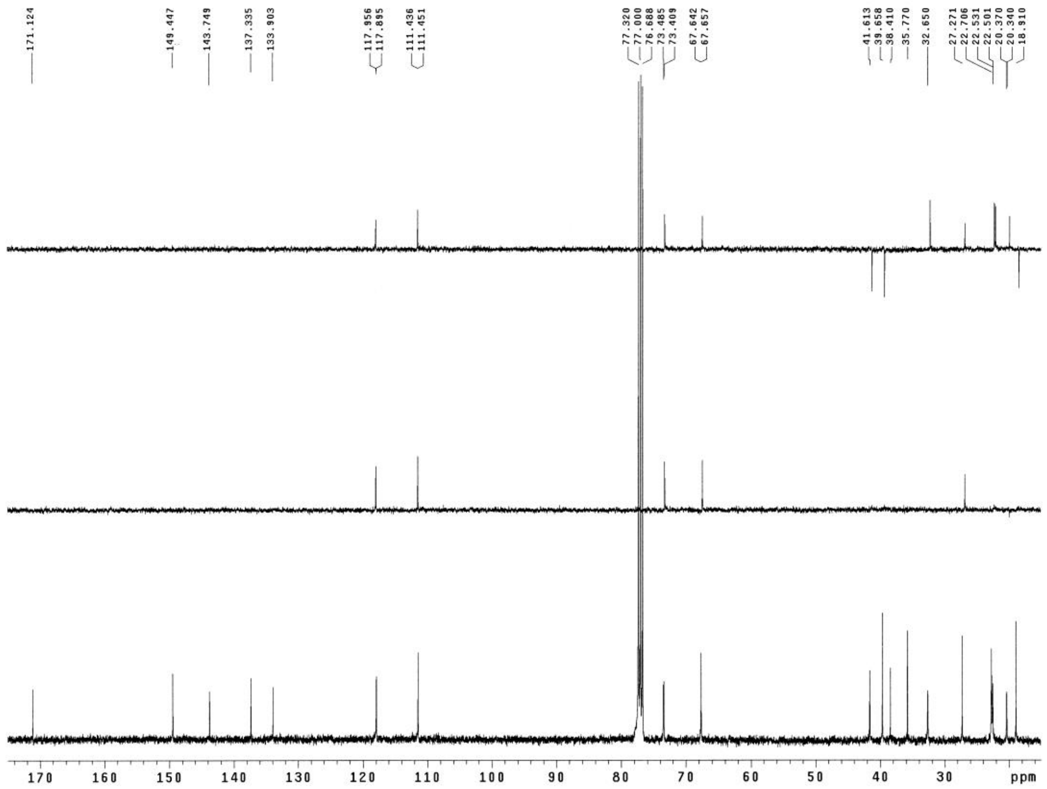

Figure S20. ^{13}C-NMR spectrum and DEPT of compound **4** in $CDCl_3$

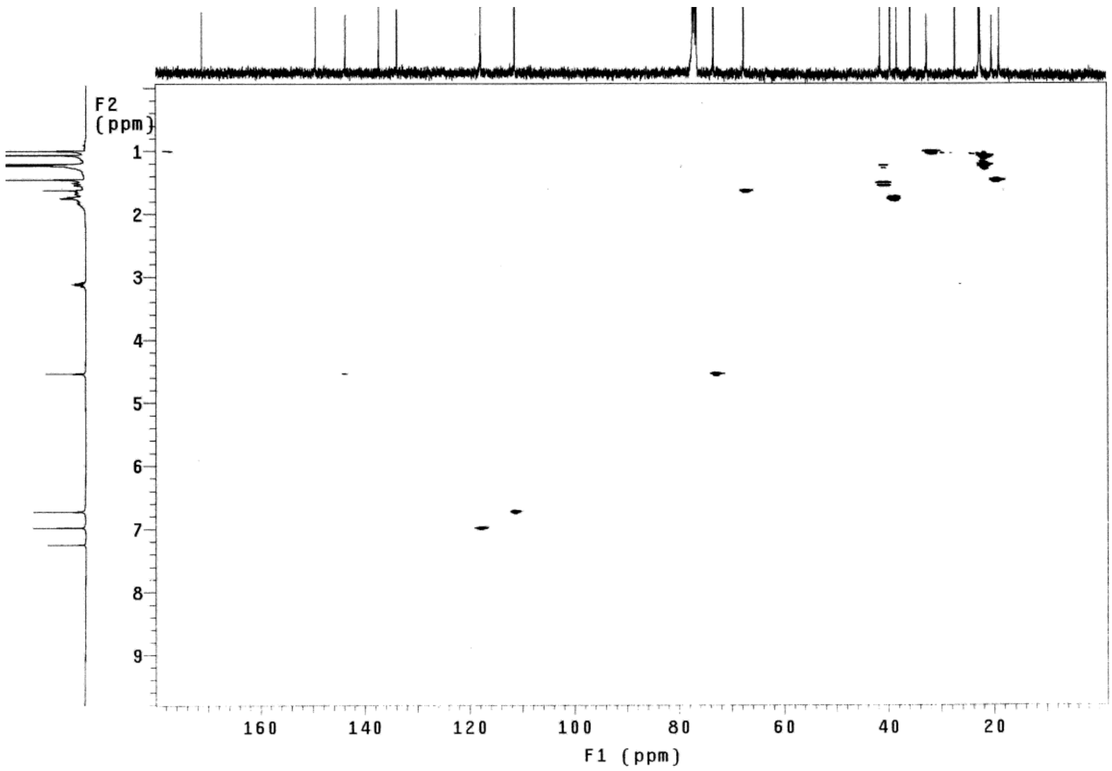

Figure S21. HMQC spectrum of compound **4** in $CDCl_3$

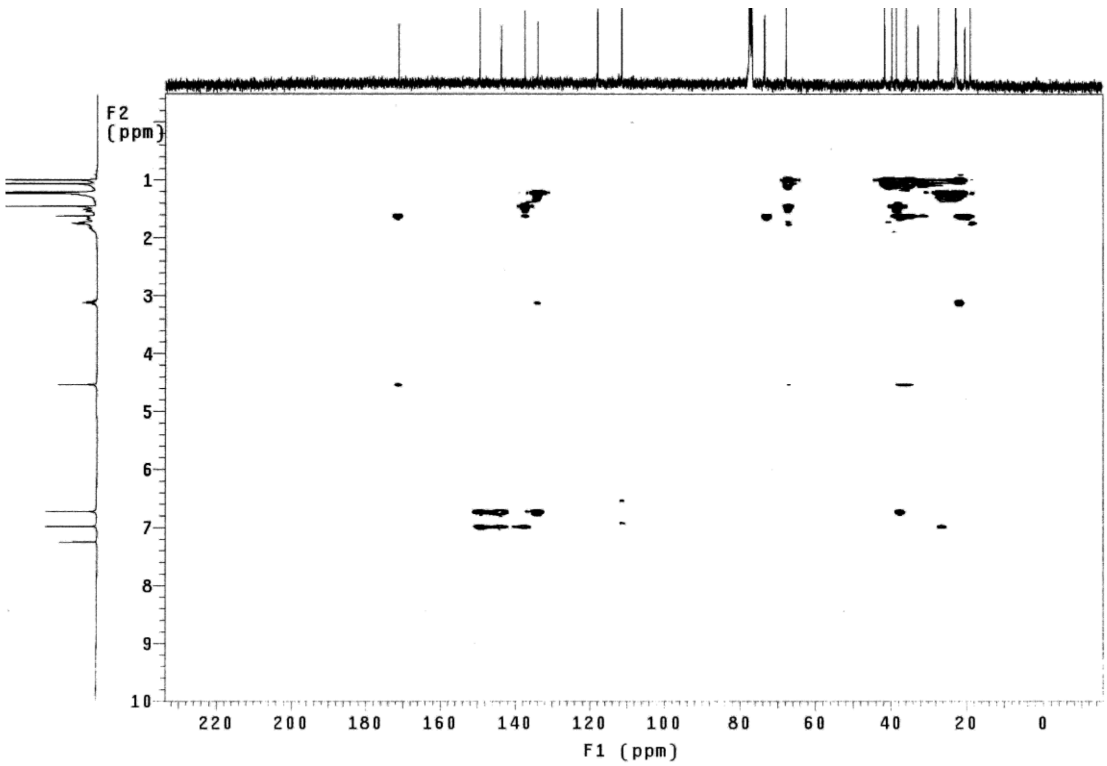

Figure S22. HMBC spectrum of compound **4** in $CDCl_3$

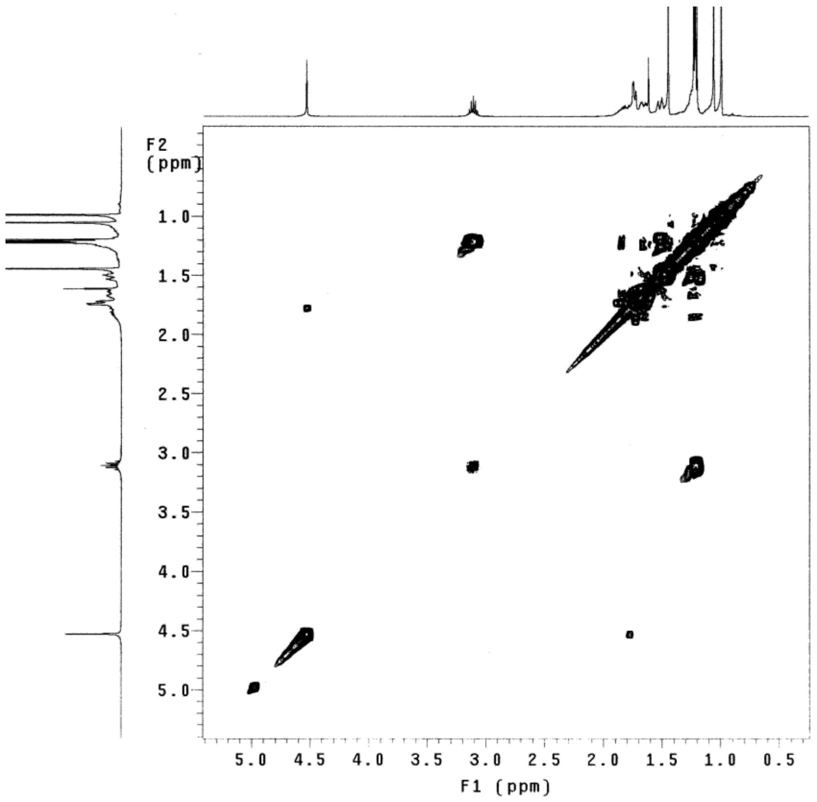

Figure S23. ^{1}H-^{1}H COSY spectrum of compound **4** in $CDCl_3$

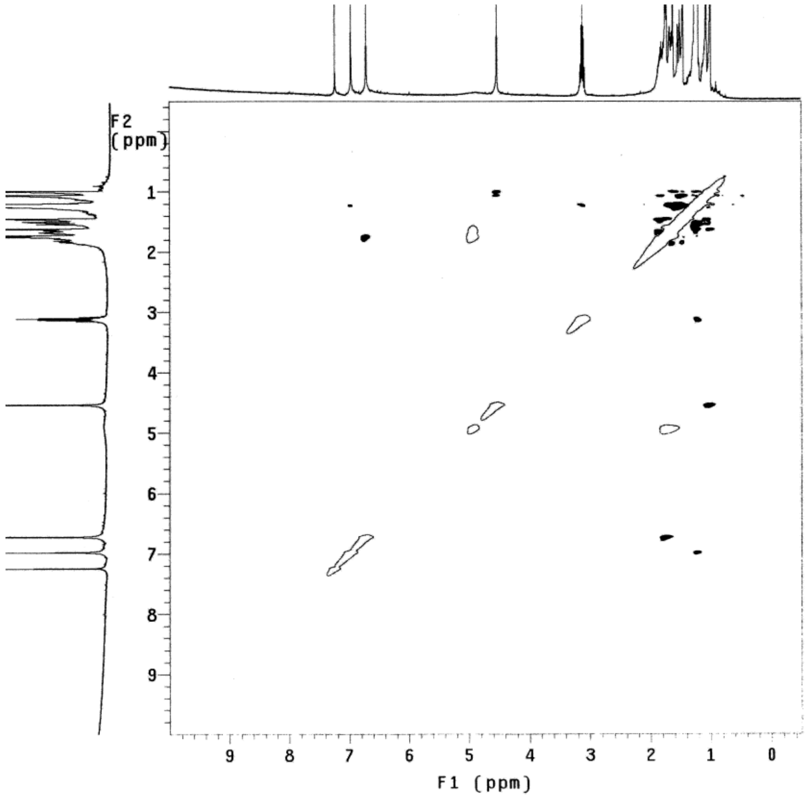

Figure S24. NOSEY spectrum of compound **4** in $CDCl_3$

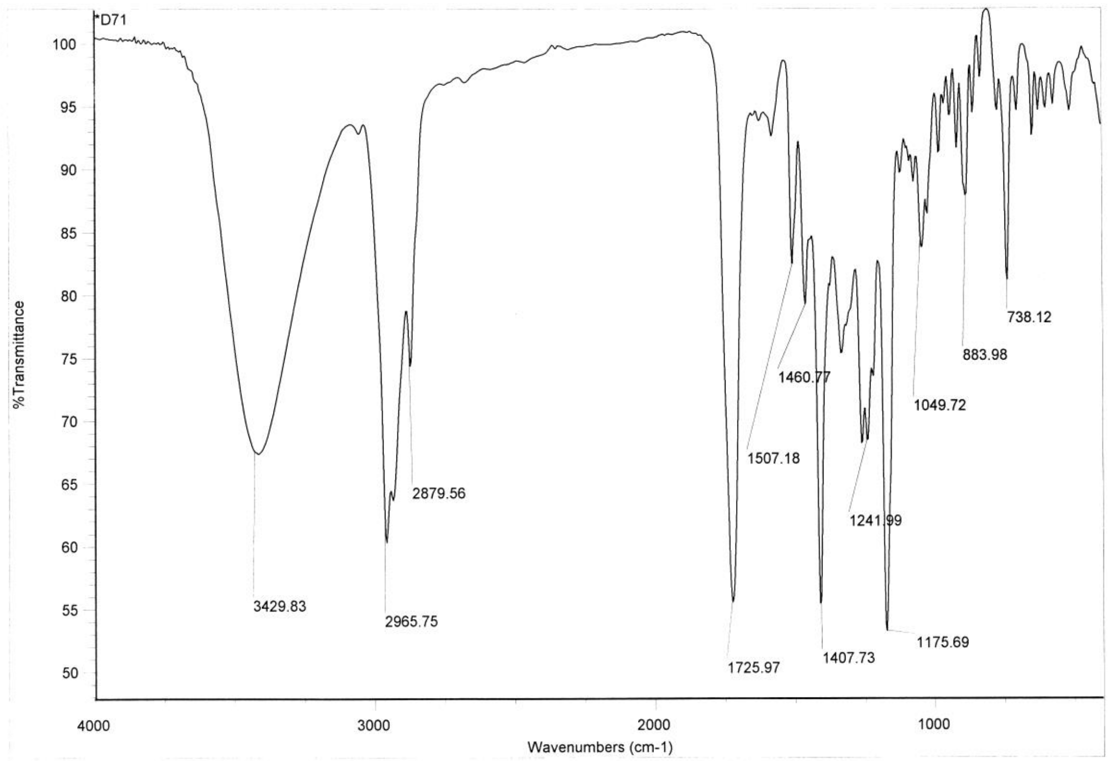

Figure S25. IR spectrum of compound **4**

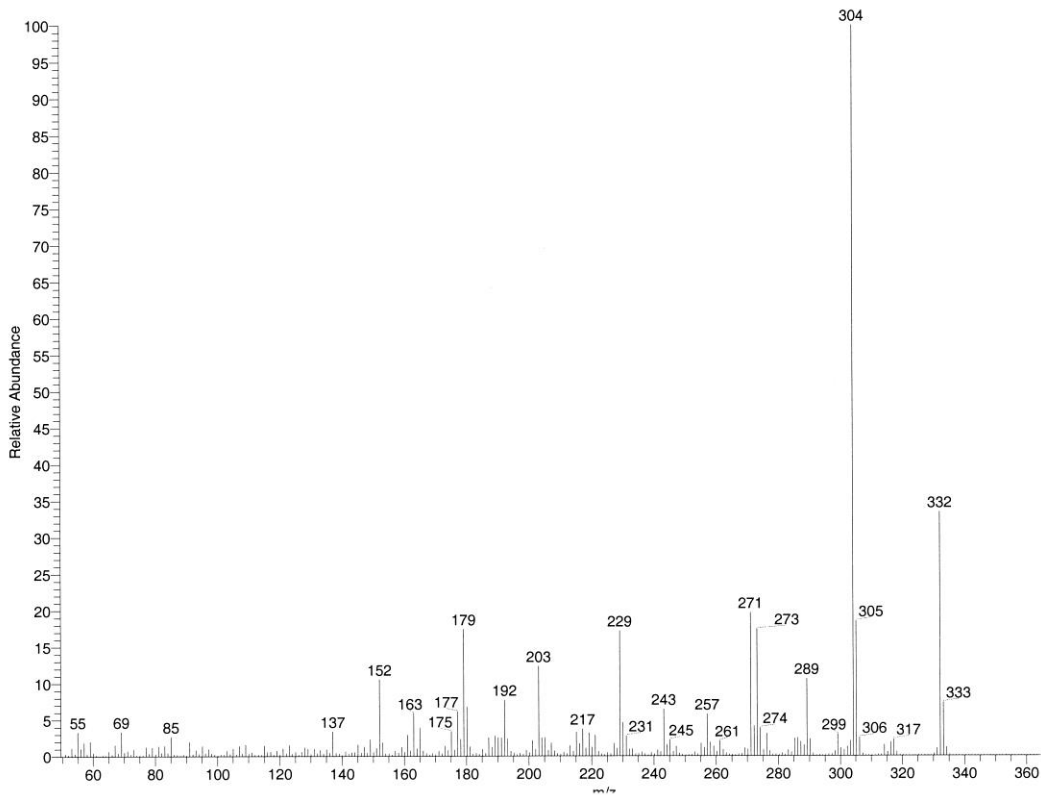

Figure S26. Mass spectrum of compound **4**

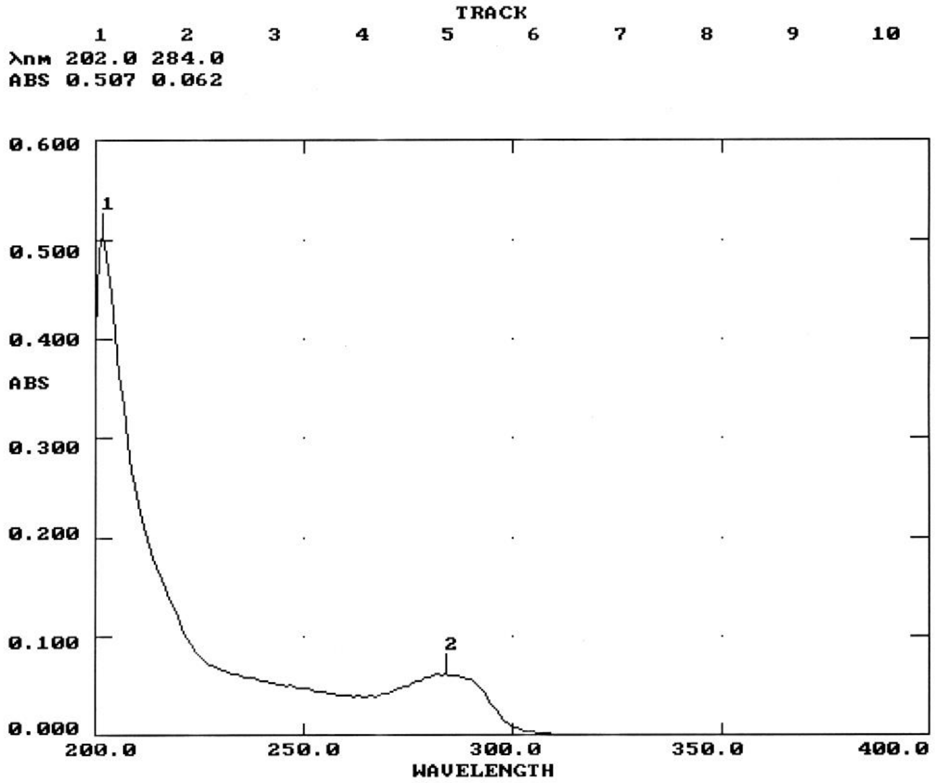

Figure S27. UV-Vis spectrum of compound **4**

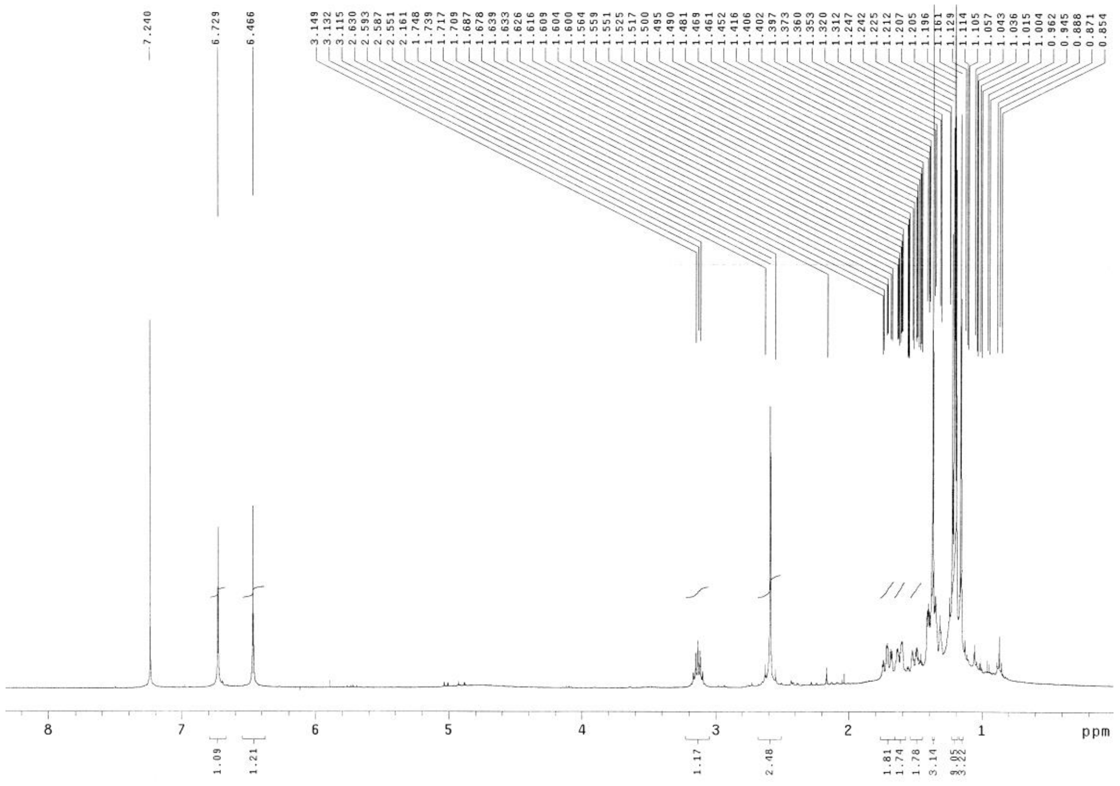

Figure S28. ^{1}H-NMR spectrum of compound **5** in $CDCl_3$

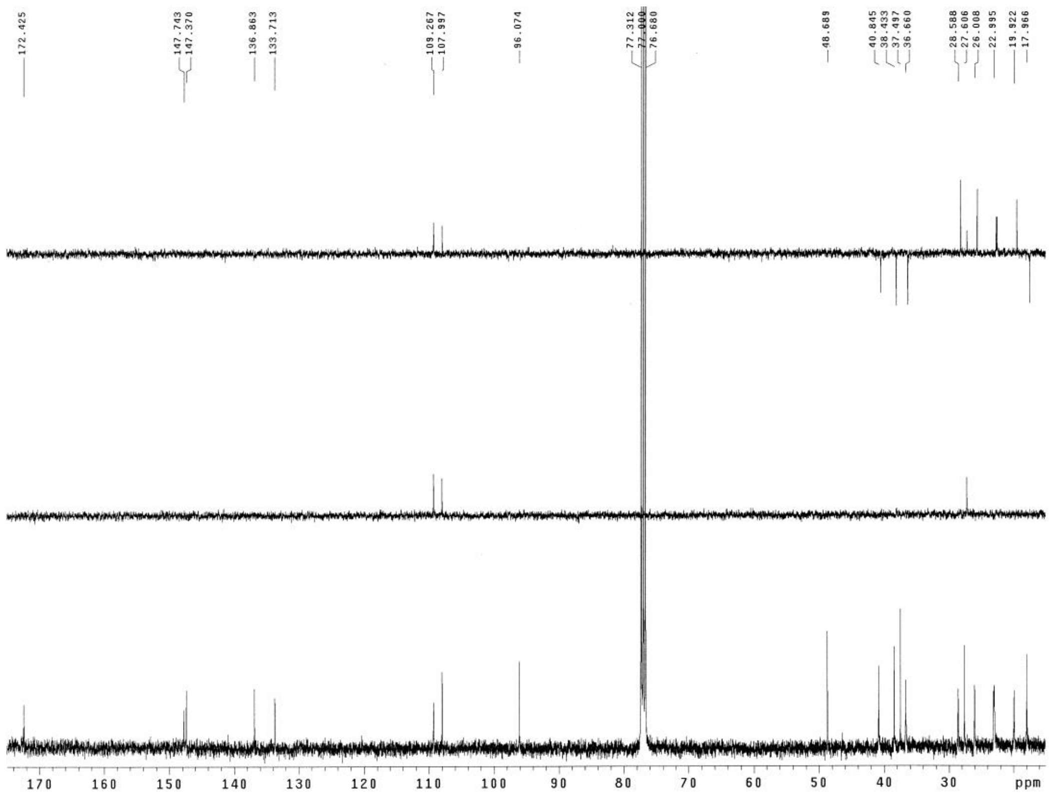

Figure S29. ^{13}C-NMR spectrum and DEPT of compound **5** in $CDCl_3$

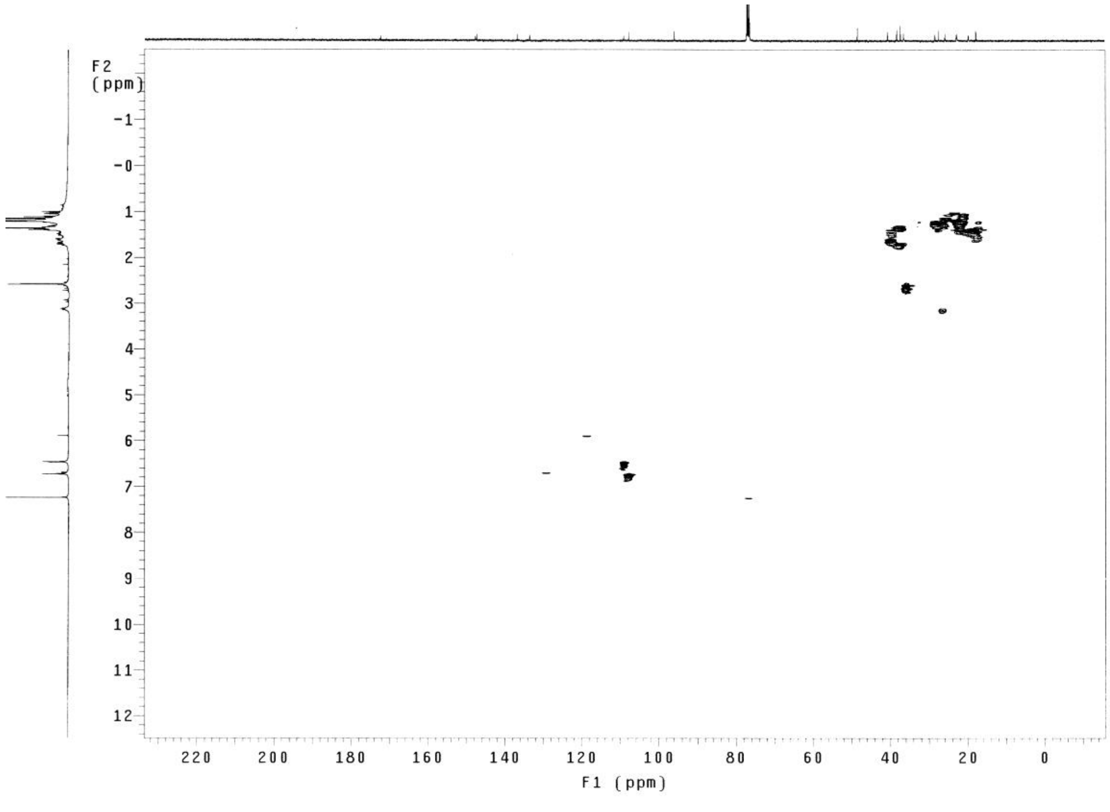

Figure S30. HMQC spectrum of compound **5** in $CDCl_3$

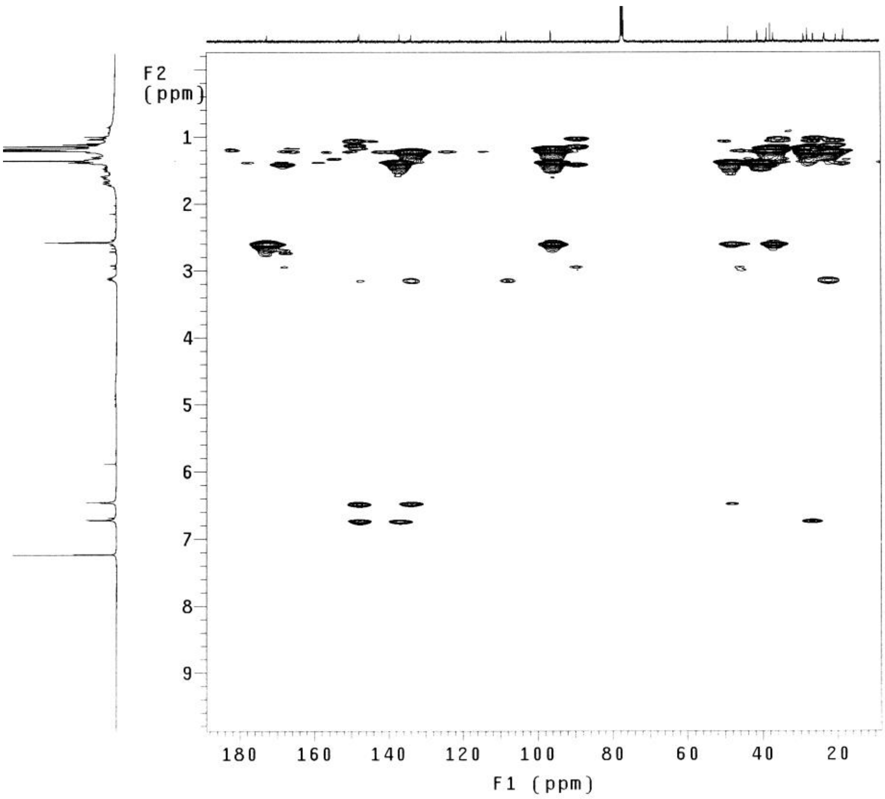

Figure S31. HMBC spectrum of compound **5** in $CDCl_3$

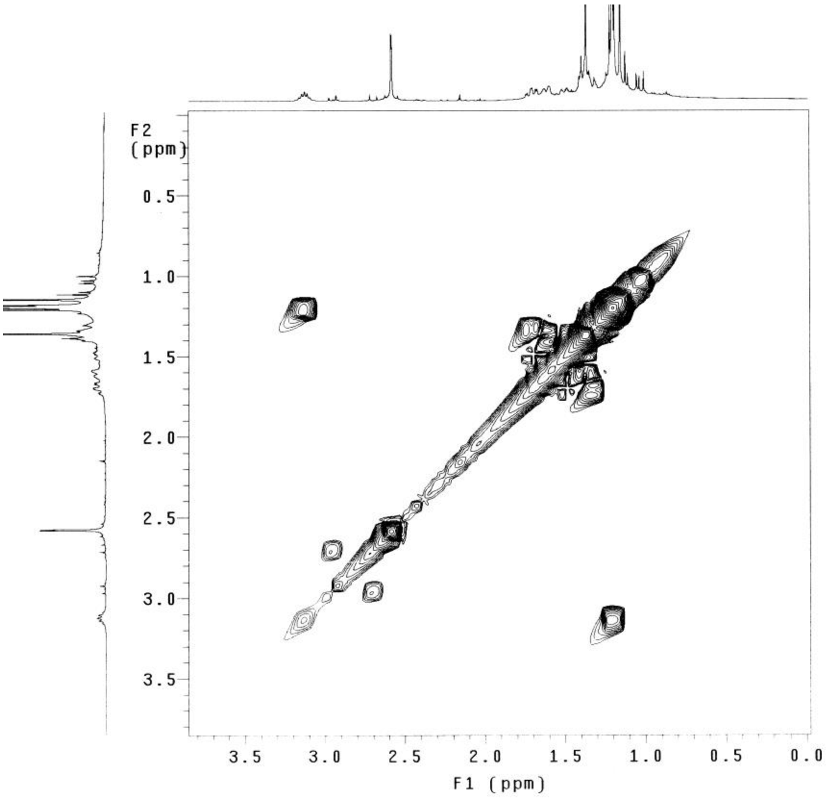

Figure S32. ^{1}H-^{1}H COSY spectrum of compound **5** in $CDCl_3$

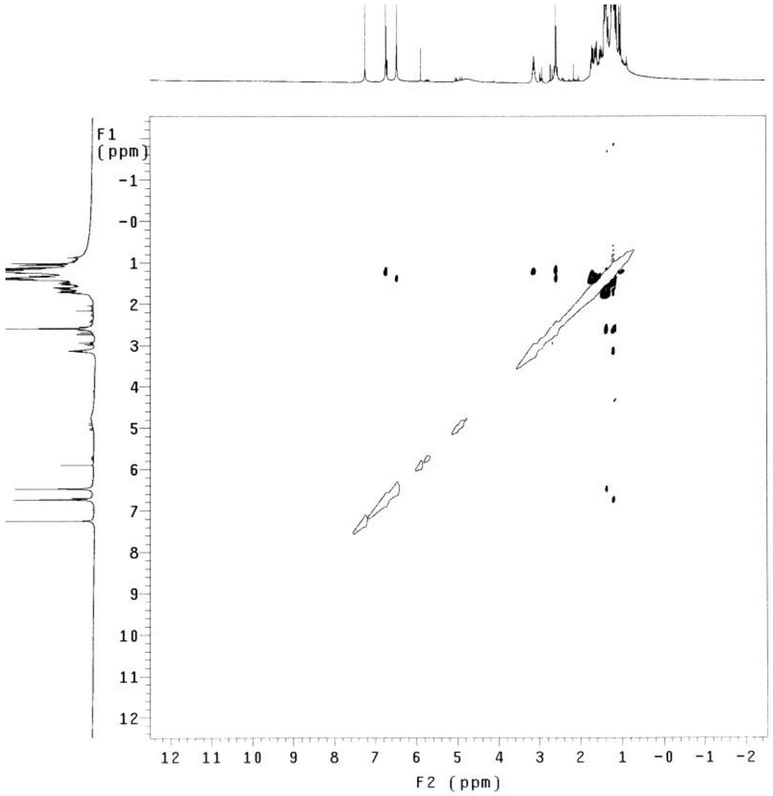

Figure S33. NOSEY spectrum of compound **5** in $CDCl_3$

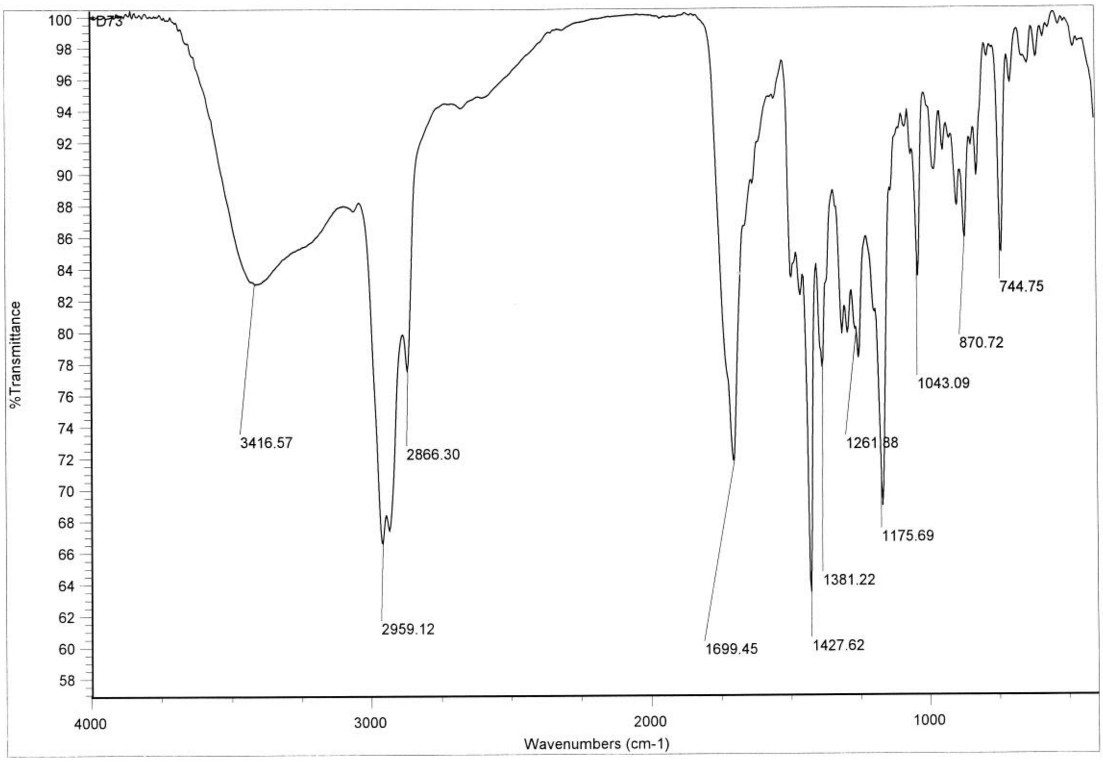

Figure S34. IR spectrum of compound **5**

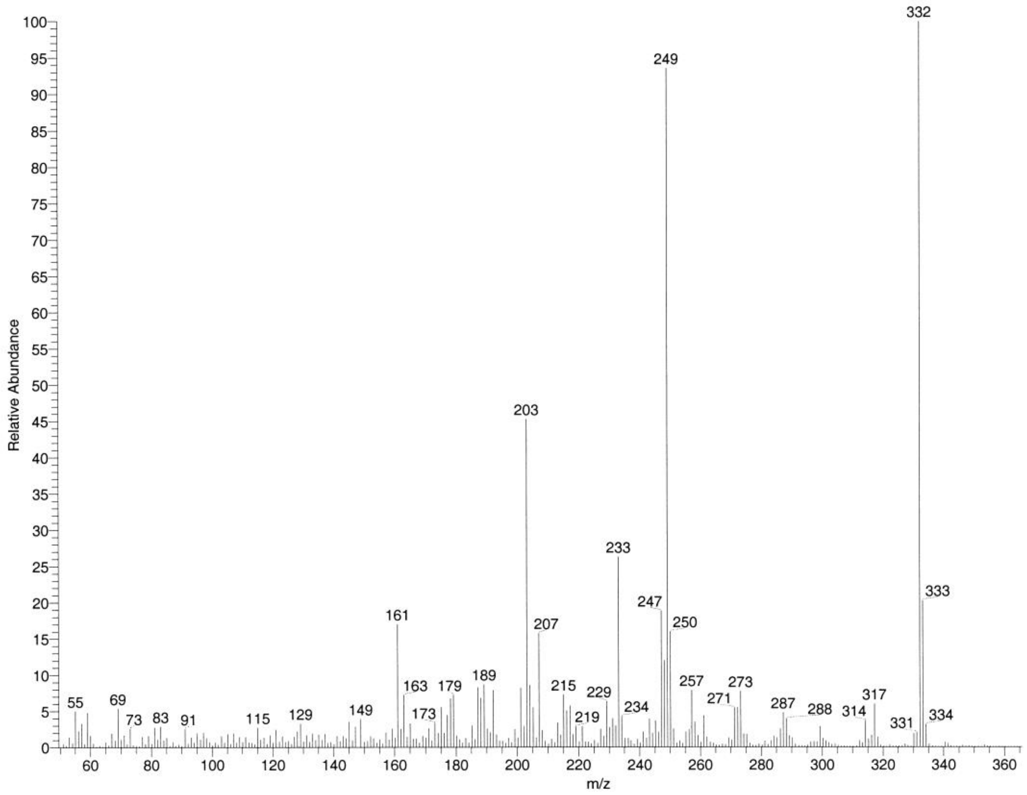

Figure S35. Mass spectrum of compound **5**

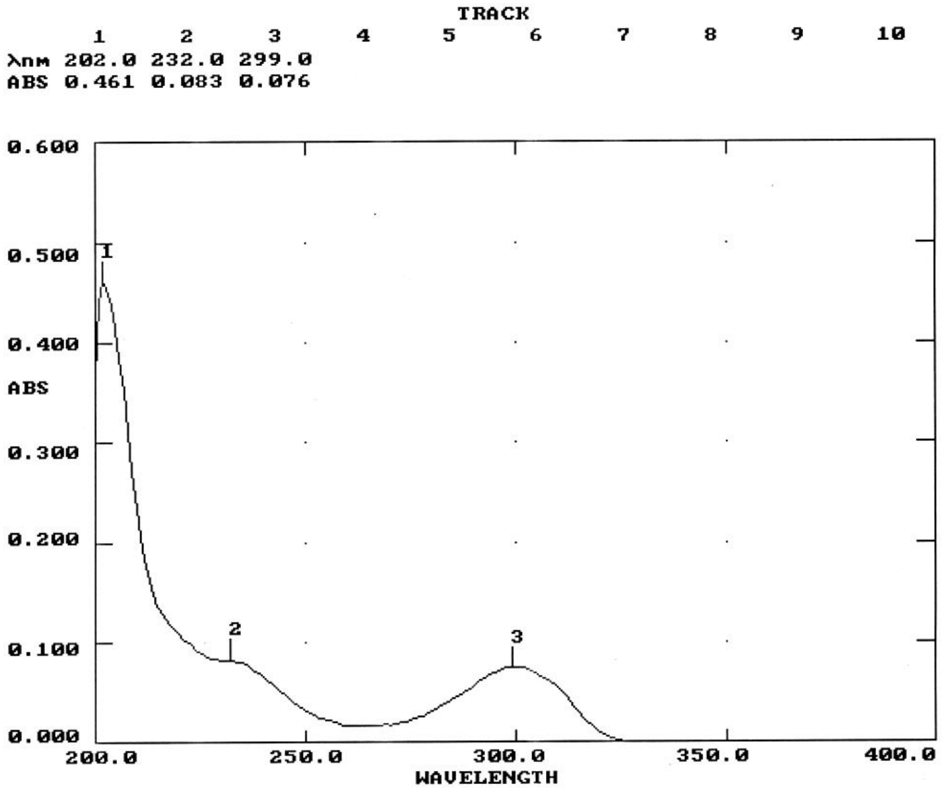

Figure S36. UV-Vis spectrum of compound **5**

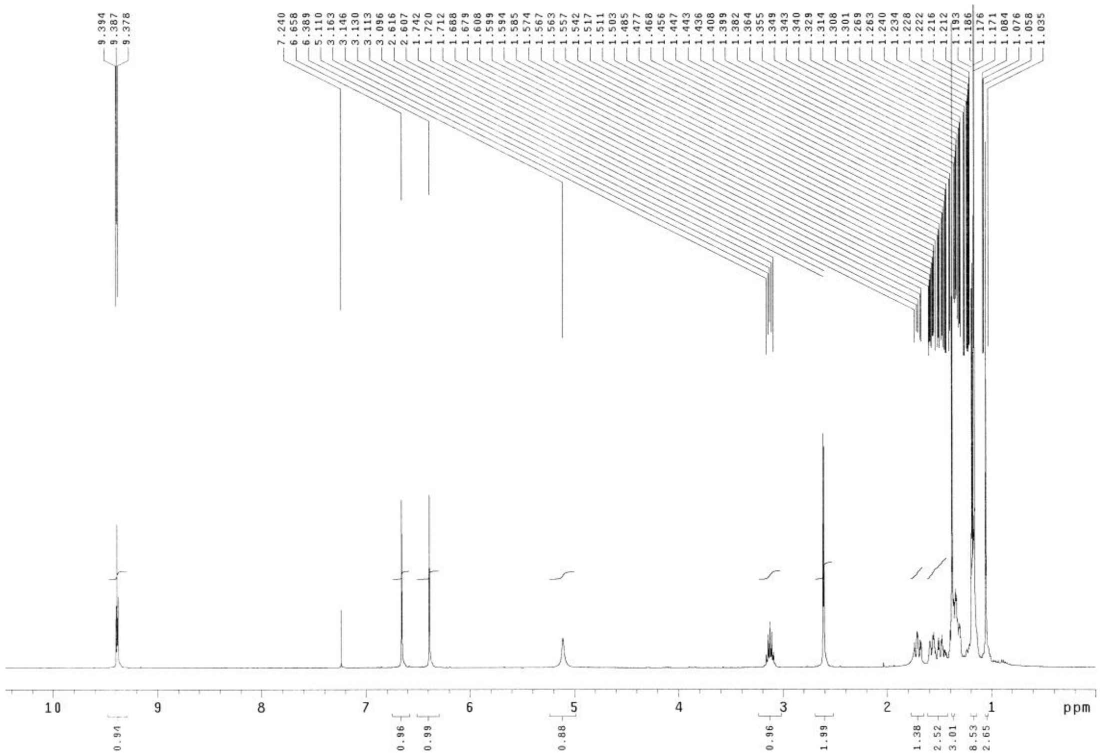

Figure S37. ^{1}H-NMR spectrum of compound **6** in $CDCl_3$

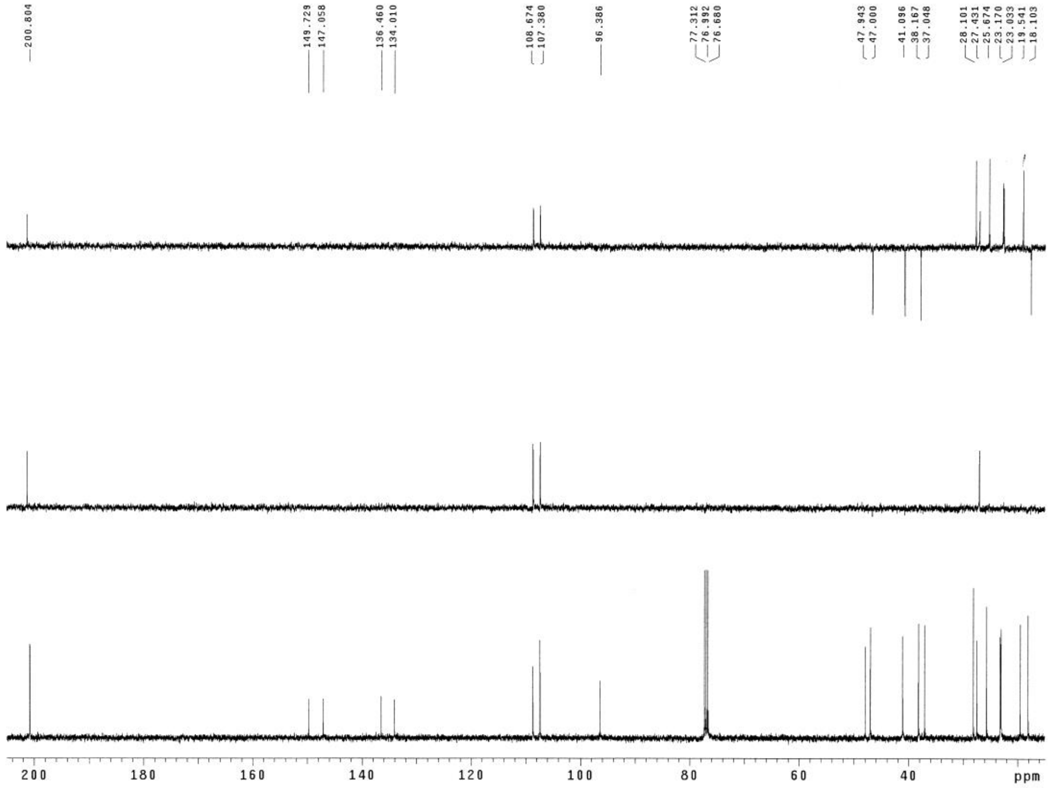

Figure S38. ^{13}C-NMR spectrum and DEPT of compound **6** in $CDCl_3$

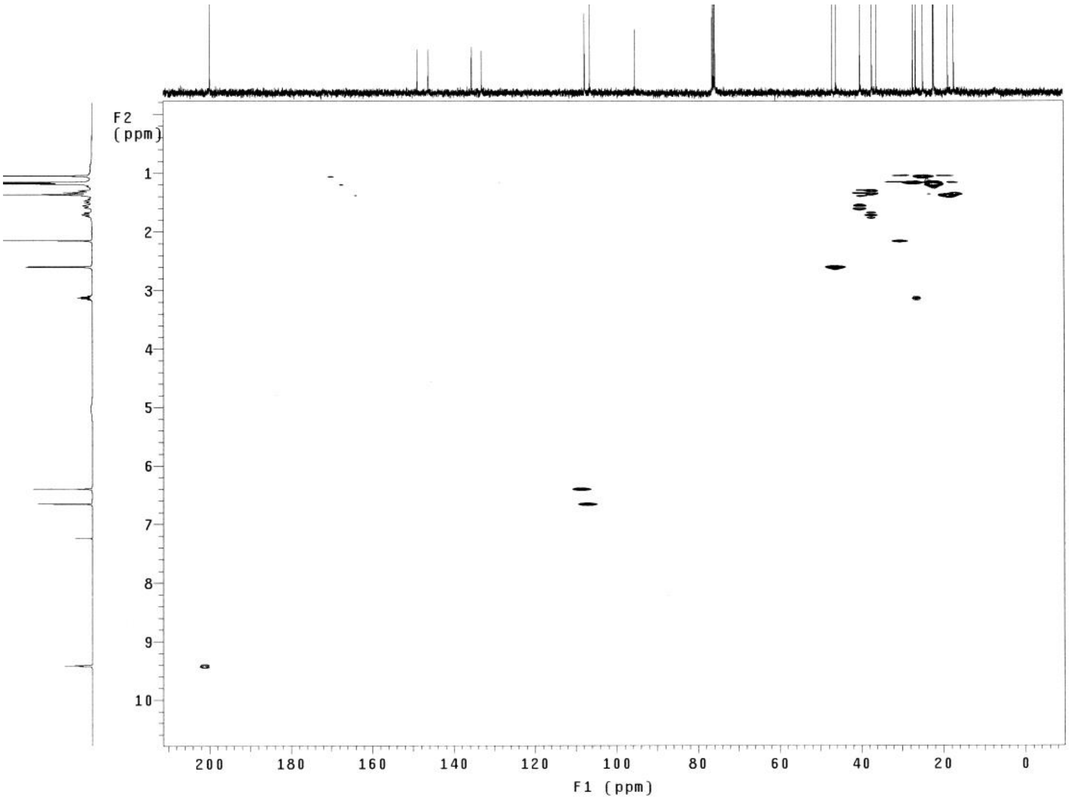

Figure S39. HMQC spectrum of compound **6** in $CDCl_3$

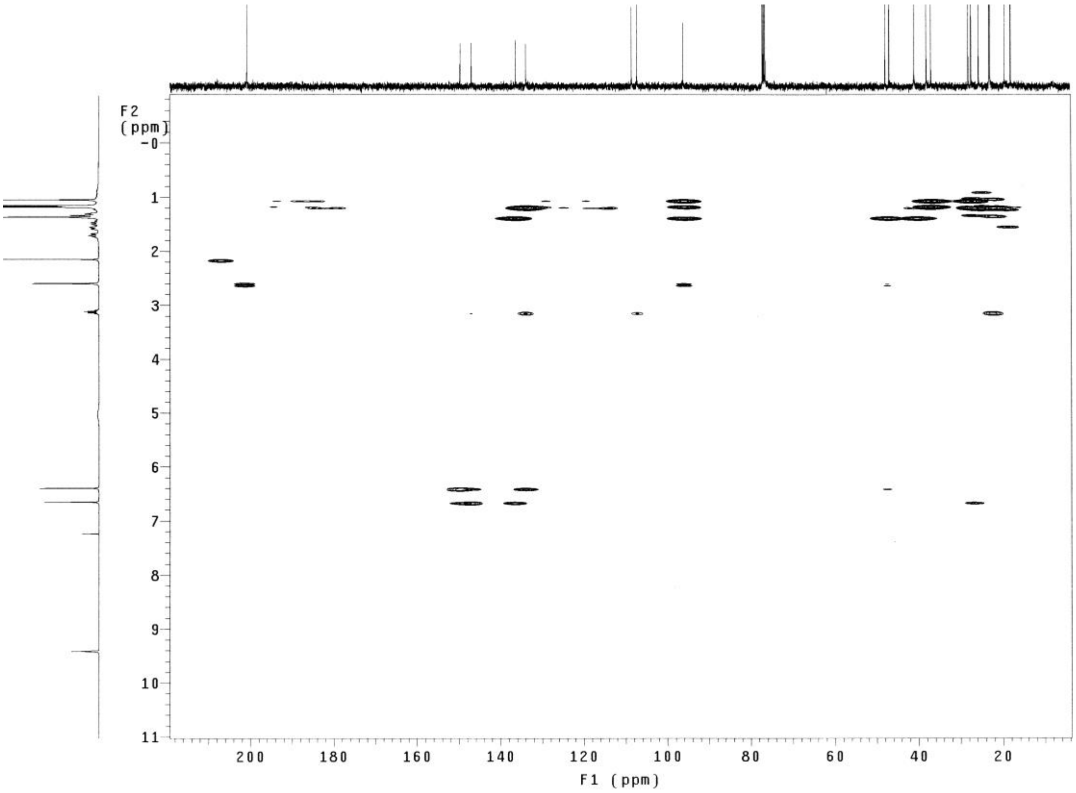

Figure S40. HMBC spectrum of compound **6** in $CDCl_3$

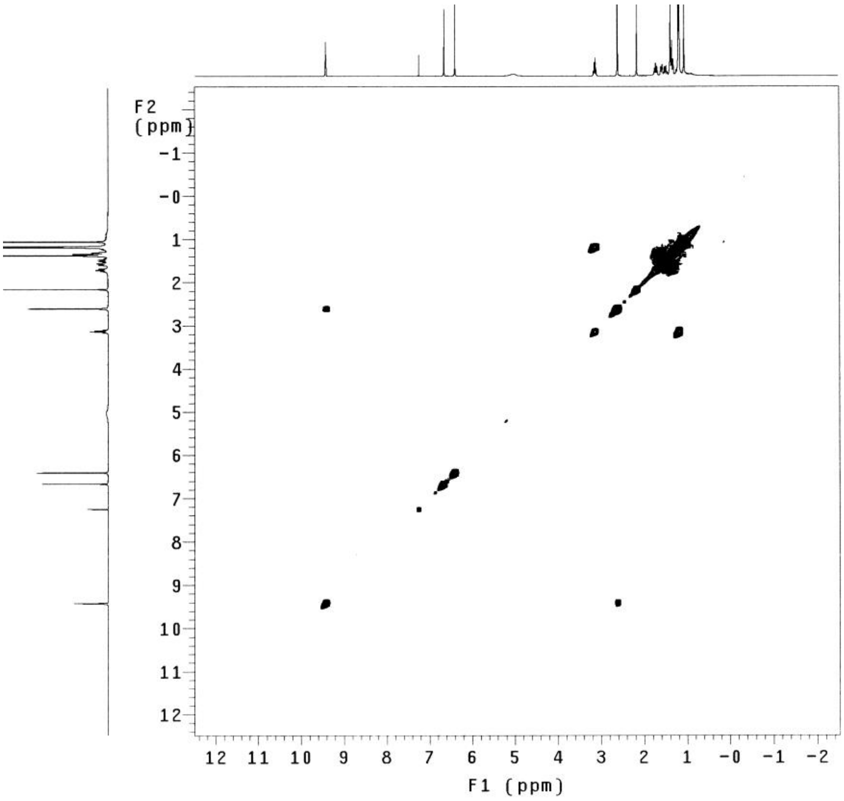

Figure S41. ^{1}H-^{1}H COSY spectrum of compound **6** in $CDCl_3$

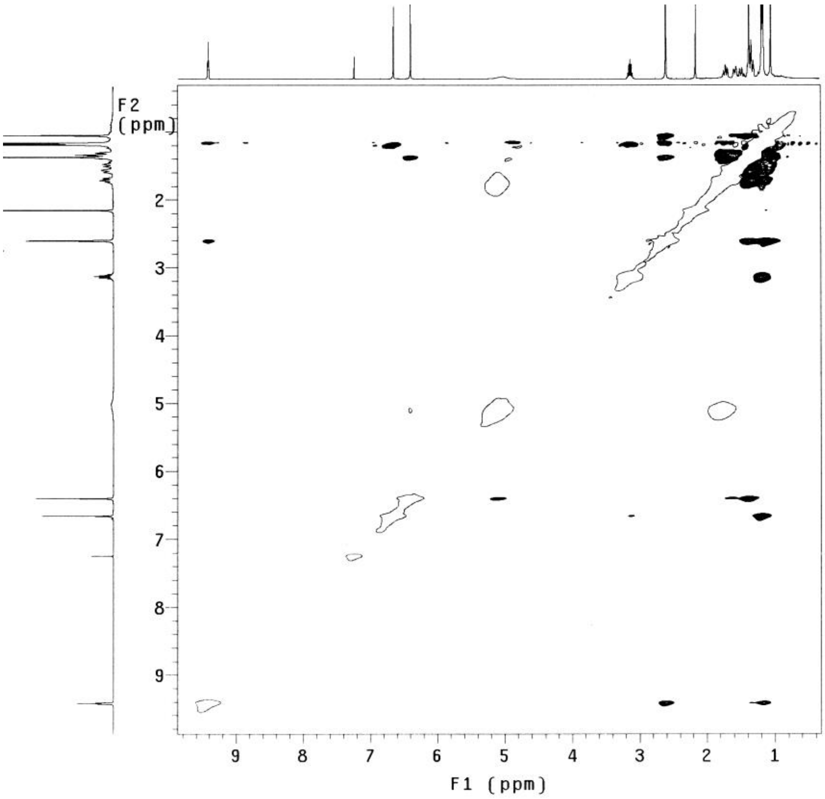

Figure S42. NOSEY spectrum of compound **6** in $CDCl_3$

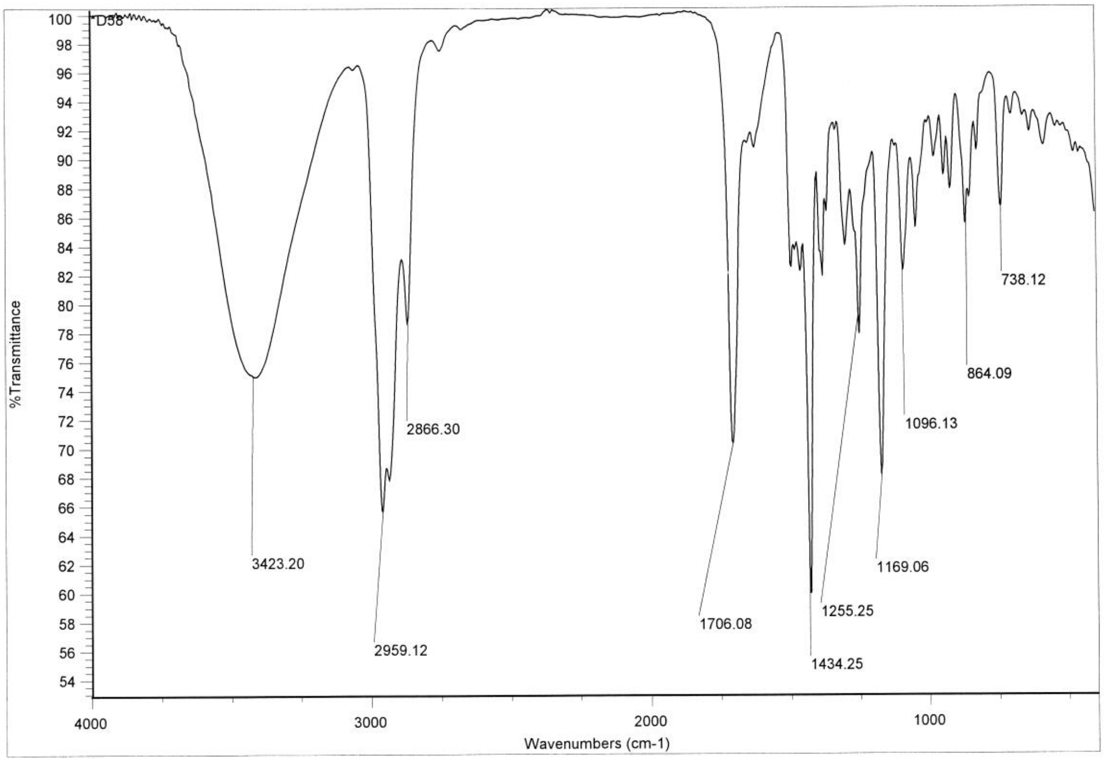

Figure S43. IR spectrum of compound **6**

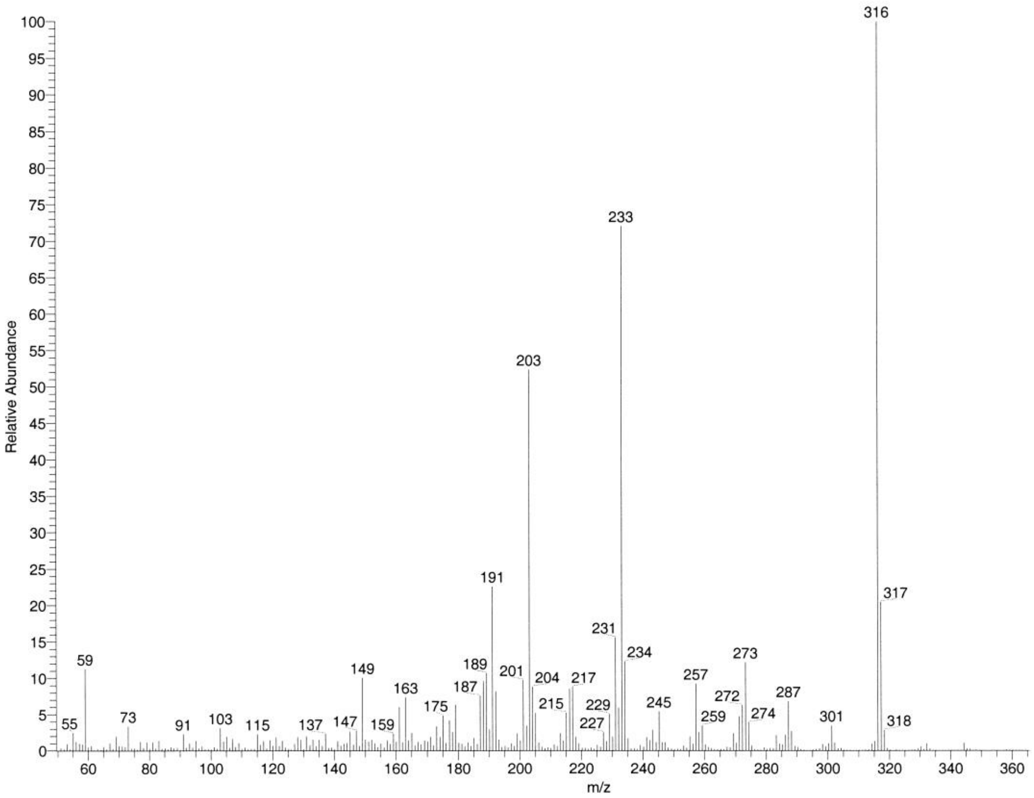

Figure S44. Mass spectrum of compound **6**

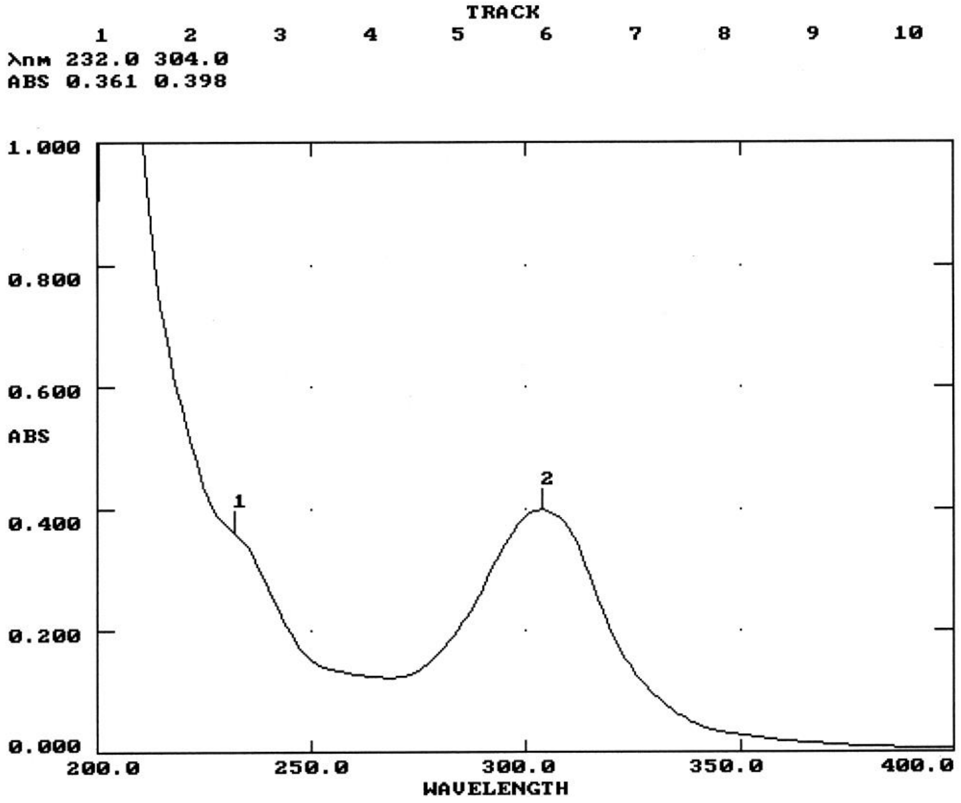

Figure S45. UV-Vis spectrum of compound **6**
